# Effect of Crumb Rubber Particle Size on the Rheological Response, Fatigue Response, and Storage Stability of Asphalt Binders: A Review

**DOI:** 10.3390/polym18182283

**Published:** 2026-09-18

**Authors:** Bhishma Oli, Sushmit Sharma Bhattarai, Hyunhwan Kim, Moon-Sup Lee, Soon-Jae Lee

**Affiliations:** 1 Department of Engineering Technology, Texas State University, San Marcos, TX 78666, USA; adm346@txstate.edu (B.O.); k_h82@txstate.edu (H.K.); sl31@txstate.edu (S.-J.L.); 2 Construction Management, Department of Construction Management, U.A. Whitaker College of Engineering, Florida Gulf Coast University, Fort Myers, FL 33965, USA; ssharmabhattarai@fgcu.edu; 3 Korea Institute of Civil Engineering and Building Technology, Goyang-si 10223, Gyeonggi-do, Republic of Korea

**Keywords:** crumb rubber modifier, particle size, asphalt-rubber interaction, storage stability, MSCR, performance-based specification

## Abstract

The use of crumb rubber modifier (CRM) from waste tires can improve asphalt-binder performance, but reported benefits vary with rubber particle size. This review evaluates particle-size effects on CRM-modified asphalt binders, covering production and morphology, swelling and asphalt–rubber interaction, rheology, fatigue, and storage stability. Studies retrieved from Scopus, Web of Science Core Collection and TRID in May 2026 were synthesized thematically. The evidence shows that particle size changes the balance between two mechanisms: coarser, swollen particles provide stronger physical reinforcement, whereas finer particles, because of their greater surface area, interact more rapidly with the asphalt phase. Finer CRM generally improves storage stability and promotes interaction, while coarser CRM can provide stronger high-temperature elastic reinforcement. These competing effects help explain conflicting findings across studies. Study-specific favorable sizes reported on a comparable basis cluster within approximately 40–80 mesh (0.180–0.425 mm, ASTM E11), but the cluster rests on a small and heterogeneous set of studies and reported optima remain dependent on binder type, rubber source, production route, rubber content, processing conditions, and performance criterion. It is therefore reported as a provisional evidence cluster, not as design guidance or a universal optimum. One direct comparison suggests that the multiple stress creep and recovery (MSCR) test may be more sensitive than G*/sinδ to particle-size-dependent high-temperature behavior. Because this rests on a single study, it is advanced here as a testable hypothesis and not as a basis for a size-sensitive specification. More consistent particle-size-appropriate testing and broader mixture- and field-scale validation are needed.

## 1. Introduction

The management of end-of-life tires represents an important global solid-waste challenge, with used tires arising at almost 14 million tonnes per year, reported as approximately 2% of total solid-waste production worldwide [1]; both quantities are expressed as mass, so the proportion is a mass fraction. Approximately 3 billion tires are disposed of annually worldwide, and this number has been projected to reach approximately 5 billion by 2030 [2]. Stockpiled and landfilled tires present fire and leaching risks, and these concerns have driven sustained efforts to recover value from scrap rubber [3]. One established recycling route is the incorporation of crumb rubber modifiers (CRM), produced by grinding used tires, into paving-grade asphalt binders. This approach combines environmental benefits with engineering improvements because, compared with unmodified binders, CRM-modified binders can improve resistance to permanent deformation, fatigue cracking, and low-temperature cracking while increasing elastic recovery and reducing temperature susceptibility [4,5]. These advantages have supported the use of CRM binders in road construction for several decades. However, the magnitude of improvement reported across studies varies considerably, and this variability is influenced not only by rubber dosage but also by the physical characteristics of the rubber itself, particularly particle size.

CRM modification involves coupled swelling, degradation, and asphalt–rubber interaction rather than simple physical dispersion. These processes produce two co-existing rheological contributions: a particle effect associated with reinforcement by undissolved swollen rubber and an interaction effect associated with modification of the continuous asphalt phase. Because these processes occur at the rubber–asphalt interface, finer CRM generally interacts more rapidly because of its greater surface-area-to-volume ratio, whereas coarser CRM tends to retain a stronger particle-reinforcement role [6,7,8]. The consequences are reflected across the full range of binder properties: reducing rubber size has been shown to alter rotational viscosity [9], high-temperature rheological properties and rutting-related parameters [10,11], multiple-stress creep and recovery and fatigue response in bio-oil-pretreated binders [12], and storage stability [13]. Particle size does not, however, act in isolation: its effects depend on rubber content and blending conditions, including interaction temperature and duration [14,15].

Reported particle-size effects are property and condition-dependent. Finer CRM is generally associated with stronger interaction and improved storage stability, whereas coarser CRM can provide stronger physical reinforcement. Closely spaced size fractions may show limited differences, and reported optima vary with rubber content, processing conditions, and test configuration. The interdependence of particle size and rubber dosage is illustrated by Chiu and Lu [16], who found that meeting the same apparent viscosity requirement under ASTM D6114 [17] required 30% coarse ground tire rubber but only 20% fine ground tire rubber. This result shows that particle size and rubber dosage cannot be selected independently.

Engineering practice, meanwhile, remains largely insensitive to rubber size. Guideline specifications for CRM particle size and gradation vary across countries and transportation agencies [4]; at the mixture scale, crumb-rubber grain size and concentration are still identified as open gaps in mix-design practice [18]; the Superpave performance-grading system was not formulated for particulate-modified binders; and even routine viscosity and grading measurements are sensitive to the test configuration adopted and to how particle size is accommodated in the procedure [19,20]. Industrial supply compounds the problem. A statistical survey of the CRM literature found that the rubber used to modify binders clusters tightly around a mean particle size of approximately 0.56 mm, with roughly 80% of reported materials finer than 0.8 mm but only about a quarter finer than 0.4 mm, reflecting a production preference for medium-size particles because finer grinding is energy-intensive and costly [21]. Recommendations that depend on very fine rubber may therefore be difficult to realize at scale, and any size guidance intended for practice must be stated against what the supply chain produces. As a result, practitioners currently lack defensible guidance on which rubber size to specify to achieve a given performance objective.

Several reviews have examined CRM-modified binders from complementary perspectives. Lo Presti [5] surveyed recycled-tire-rubber technologies and binder properties broadly; Ibrahim et al. [4] reviewed the effect of crumb-rubber addition on binder rheology, including a dedicated treatment of rubber type and particle size; Wang et al. [22] reviewed its low-temperature performance; Bressi et al. [21] combined a state-of-the-art review with a statistical analysis of fabrication parameters—among them, rubber particle size; Zheng et al. [23] reviewed rubber-asphalt compatibility; Duan et al. [24] evaluated research progress and performance of CRM asphalts; Yang et al. [25] focused on methods for improving storage stability; Lyu et al. [26] synthesized the aging evolution and sustainability implications of CRM binders; and Zakerzadeh et al. [18] critically reviewed waste tire rubber in stone mastic asphalt at the mixture scale. Particle size is acknowledged as influential throughout this body of work, is treated by Ibrahim et al. [4] as one of the internal factors governing rheology, and is quantified statistically by Bressi et al. [21]. However, a focused synthesis is still needed in which particle size is treated as the organizing variable across the full causal chain from production route and morphology, through swelling kinetics and the particle/interaction-effect balance, to high-temperature rheology, fatigue response, storage stability, size-specific recommendations, and future standardization needs. Table 1 compares the stated scope of these reviews with that of the present review.

Three differences follow from the table. First, particle size appears in earlier reviews as one factor among several; here it is the axis along which the evidence is organized, which is what makes the criterion-specific comparison in Section 7.1 possible. Second, no earlier review places reported favorable sizes on a common metric basis, and Section 2.6 shows why nominally similar mesh designations are not directly comparable. Third, the present review reports the limits of the evidence explicitly—where an interpretation is not experimentally isolated (Section 5.3), where a claim rests on a single study (Section 5.4), and where a reported optimum lies outside the proposed cluster (Section 7.1).

Accordingly, the primary objective of this review is to critically examine crumb-rubber particle size as the central variable linking production route, particle morphology and surface area, swelling and interaction mechanisms, rheological response, fatigue response, and storage stability at the binder scale. Unlike fatigue-specific reviews that follow a single distress across several evaluation scales, the present review is organized around a single material variable and examines its effects across rheological behavior, fatigue response, and storage stability, principally at the binder scale; the two organizing principles are complementary rather than overlapping. It further integrates the available evidence to identify practical particle-size ranges and the testing and standardization needs required for size-aware binder specification. The principal contribution of this review is the use of the particle-effect/interaction-effect framework to reconcile the contradictory particle-size findings and the criterion-specific compilation of reported favorable sizes, on a common ASTM E11 metric basis, into a provisional evidence cluster of approximately 40–80 mesh (0.180–0.425 mm). The review further identifies the apparent greater particle-size sensitivity of the multiple stress creep and recovery (MSCR) test relative to G*/sinδ as a hypothesis requiring independent validation.

The primary focus of this review is the binder scale, particularly CRM-modified asphalt binders for which particle size or gradation is reported. Mixture-scale performance studies and chemical or additive-based stabilization studies are considered only when they provide direct supporting context for interpreting binder behavior, particle-size effects, or practical specification needs. The review first examines CRM production, morphology, surface area, and interaction mechanisms, followed by particle-size effects on rheological performance and storage stability. The final section sets out what the evidence does and does not support on particle-size selection, together with priorities for future standardization.

## 2. Review Methodology

### 2.1. Design and Reporting Basis

This review is a structured narrative synthesis, not a systematic review. It follows the identification, screening, and selection logic of the Preferred Reporting Items for Systematic Reviews and Meta-Analyses (PRISMA) statement for transparency of study selection, but does not include meta-analysis, pooled estimation, or formal risk-of-bias scoring. This distinction is stated explicitly because the heterogeneity of binders, rubber sources, production routes, dosages, test protocols, and performance criteria across the primary literature precludes quantitative pooling. No formal study-quality instrument was applied. Instead, evidence limitations were appraised descriptively through the distinction between primary and contextual evidence, whether particle size was isolated experimentally, the completeness of reported test conditions, and the comparability of particle-size descriptors. Where the strength of the underlying evidence is limited, this is stated in the text rather than absorbed into a summary judgment.

### 2.2. Search Strategy

Searches were executed in May 2026 in three databases. The coverage period was not restricted at the search stage; the earliest included record dates from 1992 and the most recent from 2026. The core string combined three concept blocks:

(“crumb rubber” OR “ground tire rubber” OR “GTR” OR “tire rubber” OR “scrap tire rubber”) AND (asphalt OR bitumen OR binder) AND (“particle size” OR gradation OR mesh OR “size distribution”).

Four supplementary searches targeted the principal themes of the review: interaction mechanisms (swelling, degradation, dissolution, devulcanization); storage stability (phase separation, segregation); rheological and fatigue response (multiple-stress creep and recovery, linear amplitude sweep, rutting, fatigue); and production and morphology (ambient, cryogenic, grinding, surface area). Database searches were complemented by backward and forward citation tracking of included studies and by hand-searching of venues in which foundational work on rubberized binders appears, including Transportation Research Record, the Asphalt Rubber conference series, and the Journal of the Association of Asphalt Paving Technologists.

### 2.3. Eligibility Criteria

Mixture-scale and specification-oriented studies were not treated as primary evidence. A limited number were retained as supporting context under a single explicit rule: the study is cited only where it (i) interprets or constrains a binder-scale finding already established from primary evidence, or (ii) documents a specification or practice position that the binder-scale evidence must eventually serve. Such studies are identified as supporting context at the point of use and are excluded from the size-versus-property synthesis in Section 7. This distinction is applied consistently in the study-level tables of Section 5, Section 6 and Section 7.

### 2.4. Screening, Selection, and Reproducibility

The searches were first executed in May 2026. A stage-by-stage record of the numbers screened and excluded at that time was not maintained, and this review does not reconstruct one: presenting an undocumented cascade as though it had been recorded would misrepresent how the review was carried out.

Reproducibility is instead established prospectively. The strategy in Table 2 is stated in full, was re-executed on a stated date, and the counts it returns on that date are reported alongside it. A reader can therefore repeat the search and compare their yield with ours directly—which a historical cascade, being unrepeatable, would not permit. On re-execution the three databases returned 1005 records in total before de-duplication (Table 2); this figure is the yield of the stated strategy on the stated dates and is not a record of the May 2026 screening cascade.

The 83 sources retained are listed individually in Supplementary Table S1, which records for each one its source type, whether its study-level detail is tabulated in the study-level tables of Section 5, Section 6 and Section 7, and whether a source—an abstract or the full text—was obtainable for citation-accuracy checking. All 83 are cited in the text; numbering follows order of first appearance, and the reference list additionally contains the five standards cited in the text.

Records were de-duplicated, screened by title and abstract against the criteria in Table 3, and then assessed in full text against the same criteria. Screening was carried out by one reviewer. The resulting inclusion and exclusion decisions were then reviewed by two of the authors, who adjusted them by agreement; where the two did not initially agree, the record was discussed until a common decision was reached. Because the initial screening was performed by a single reviewer, no inter-screener disagreement arose at that stage, and the two-author review served as the check on single-reviewer error.

### 2.5. Data Extraction

For each included study the following were extracted where reported: CRM source and production route; particle size or gradation and the basis on which it is expressed; binder grade; rubber dosage and dosage basis; blending temperature, duration, and shear; aging condition; test method, geometry, and temperature; response variable; and principal finding. Fields not reported in the source are shown as “not reported” rather than inferred.

### 2.6. Particle-Size Terminology and Unit Conventions

Mesh designations are sieve-opening labels, not particle diameters, and they are not interchangeable across standards. Throughout this review, mesh numbers refer to ASTM E11 sieves and the corresponding nominal openings in Table 4 are given alongside every mesh value. The provisional evidence cluster discussed in Section 7 therefore corresponds to 0.180–0.425 mm.

Four size descriptors appear in the primary literature and are not equivalent. They are distinguished throughout this review and identified for every study in the study-level tables of Section 5, Section 6 and Section 7:

- Passing size—material passing a stated sieve (an upper bound; for example “≤0.5 mm”).
- Retained fraction—material between two sieves (for example 30–40 mesh, 0.425–0.600 mm).
- Mean or nominal diameter—a single representative value, usually without a stated distribution.
- Full gradation—a reported particle-size distribution across several sieves.

Two consequences follow. First, a “40 mesh” material in one study need not be comparable with a “40 mesh” material in another: Lei et al. [12] report 40 mesh as 0.38 mm, whereas the ASTM E11 opening for No. 40 is 0.425 mm, indicating a different sieve series or a nominal manufacturer designation. Second, an upper-bound descriptor and a retained fraction cannot be placed at the same point on a size axis. Where a source does not state which descriptor it uses, this is recorded as “basis not stated” rather than assumed.

A further distinction applies throughout Section 4 and Section 6: the initial CRM size charged to the blend differs from the residual size after swelling, degradation, and partial dissolution. The two are identified separately wherever the source permits, because storage stability and the particle/interaction balance respond to the residual state rather than to the charged size.

## 3. CRM Production Methods, Particle Morphology, and Surface Area Effects

**Figure 1:**
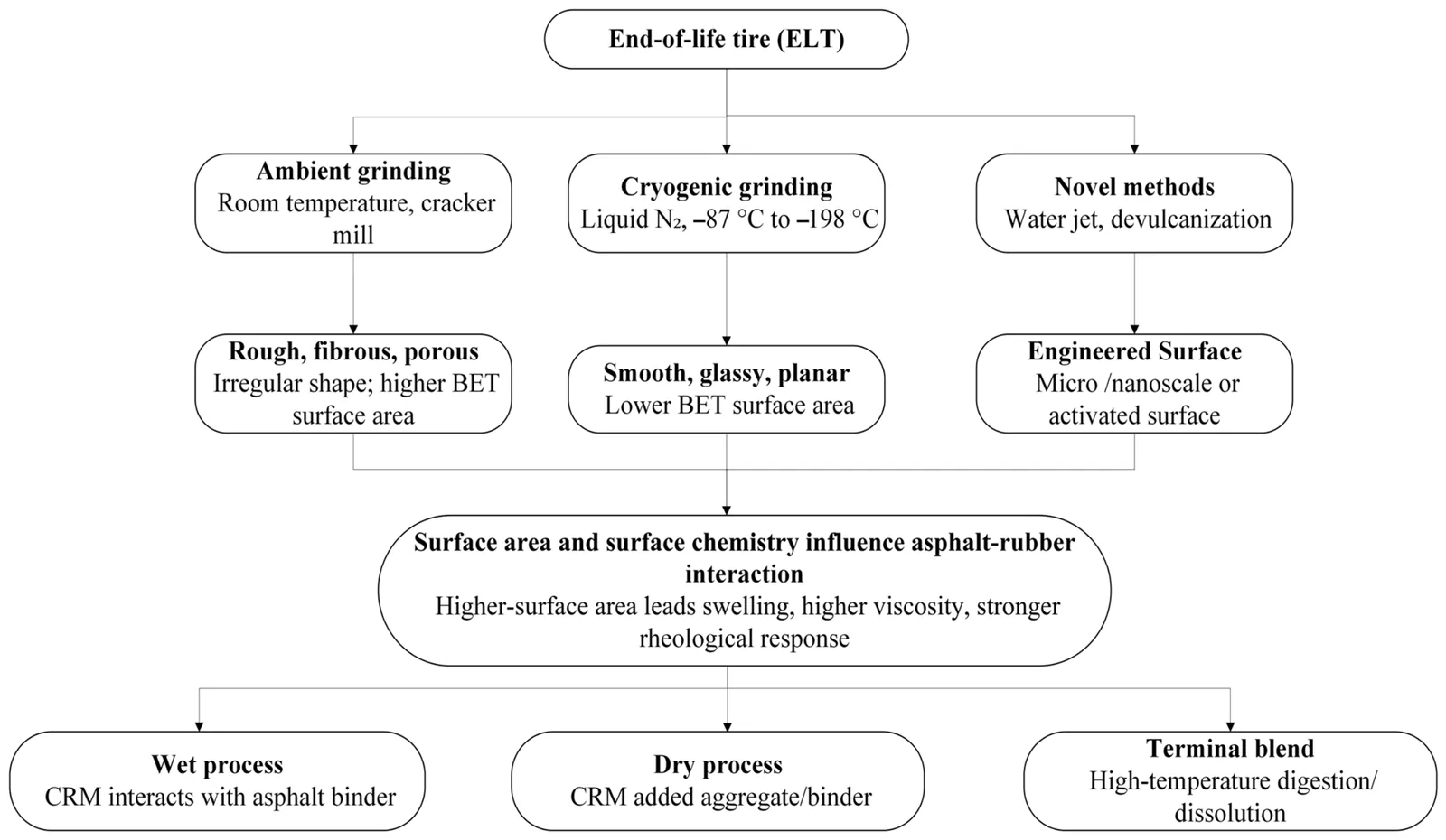
Conceptual framework linking CRM production route, particle morphology, specific surface area, and asphalt–rubber interaction. CRM, crumb rubber modifier. The relationships shown are the authors’ synthesis of the sources cited in Section 3; the figure is conceptual and reports no measured quantity.

Crumb rubber modifier (CRM) is derived from end-of-life tires (ELTs) through size reduction processes that fundamentally determine particle shape, surface texture, and specific surface area; the three physical characteristics most directly governing binder modification effectiveness [11,15,28]. The two dominant industrial methods are ambient grinding and cryogenic grinding, which produce particles of contrasting morphology and surface activity [11,15,29,30]. Early state-of-practice guidance already distinguished the production routes by the particles they yield, the cracker mill giving irregular, torn particles of large surface area and the granulator giving cubical, cut particles of low surface area, and identified surface area as the control on the rate of reaction with the asphalt cement [31]. In ambient grinding, tires are shredded at room temperature in cracker mills; the tearing mechanism yields irregular, fibrous, porous particles with rough surface asperities [29,32]. Cryogenic grinding cools rubber to −87 °C to −198 °C with liquid nitrogen before hammer milling; brittle fracture produces smooth, glassy, planar particles of lower specific surface area [15,29,30]. Emerging routes, such as high-pressure water-jet processing and rubber devulcanization, extend this range further. Water-jet cutting yields surface roughness exceeding that of ambient grinding [28], while devulcanization reduces particles to the micro–nano scale with altered surface chemistry [33]. In addition to these size-reduction routes, post-processing surface activation can act on the rubber without substantially changing the bulk particle size, although the reported mechanisms differ. Bio-based functionalization grafts biomolecules onto the rubber surface, altering its surface chemistry [34], whereas microwave activation has been reported to proceed mainly through physical changes, with the subsequent swelling reaction becoming more efficient [35]. Figure 1 summarizes the production pathway from ELT through grinding method, morphology, and surface area to blending process selection.

Because these production methods generate different particle morphologies, the resulting differences in surface area, texture, and size distribution become important factors controlling asphalt–rubber interaction and binder modification.

Surface area and particle morphology are among the CRM characteristics most directly associated with binder modification intensity. Experimental evidence indicates that ambient-ground CRM can exhibit approximately twice the Brunauer–Emmett–Teller (BET) surface area of cryogenic CRM at equivalent mesh size, contributing to differences in high-temperature rheological response. However, tire source, particle morphology, and size distribution can produce substantial differences even among CRM materials with similar nominal gradations, demonstrating that nominal particle size alone is insufficient to characterize CRM modification behavior [11,15,36]. In a comparison of eleven commercial products, surface area and particle size accounted for most of the variation in binder response, whereas grinding temperature had little independent influence once surface area was taken into account, because a finely ground cryogenic product carried more surface area than the coarser ambient-ground materials [36]. Singh et al. [8] showed that CRM modifies asphalt through both physical reinforcement from rubber particles and asphalt–rubber interaction caused by absorption and swelling. The balance between these two effects depends on rubber particle size and testing condition. These particle-level differences become especially important during blending because wet, dry, and terminal-blend processes provide different opportunities for swelling, interaction, and dissolution.

The three principal blending processes each interact differently with particle morphology. In the wet process (175–200 °C), CRM particles blended directly into liquid asphalt swell by absorbing the maltenic fraction; ambient CRM achieves higher viscosity and G^*^ than cryogenic at equivalent content due to its greater surface-area-driven absorption capacity [14,29,32,37]. Jeong et al. [14] demonstrated that blending time, temperature, and CRM content must be optimized because interaction conditions strongly affect viscosity and high-temperature rheological properties. Later, Ren et al. [38] showed that finer CRM particles reach swelling equilibrium earlier and enter the degradation regime earlier than coarser particles.

In the dry process, coarser particles (0.4–9.5 mm) act as aggregate substitutes with limited binder interaction; Rath et al. [39] showed that surface pre-treatment of dry-process ground tire rubber (400–600 µm) improved the continuous high-temperature performance grade by approximately 10–12 °C, demonstrating that surface chemistry can compensate for limited interaction time [39]. In the terminal blend (TB) process (200–260 °C), particles dissolve progressively into the asphalt matrix; Huang et al. [40] confirmed via gel permeation chromatography (GPC) that CRM degradation under TB conditions redistributes rubber-chain polymers, asphaltenes, and maltenes into the continuous phase, with Wen et al. [41] finding that rubber content dominates TB storage stability while particle size effects are source-dependent [41,42]. Liang et al. [43] showed through microstructural observation that finer CRM particles produce more compatible and interpenetrating rubber-asphalt structures, whereas coarser particles tend to form discrete rubber-rich domains that are more prone to phase separation. Similar particle-size, compatibility, and phase-separation effects have also been reported or reviewed by Jamal et al. [44], Navarro et al. [10], and Zheng et al. [23]. Bahia and Davies [45] and Peralta et al. [46] demonstrated that production process is a major source of cross-study variability in CRM binder performance, with mutual chemical changes reaching equilibrium states governed by rubber-to-asphalt ratio and surface contact area rather than particle size alone [45,46,47]. The main CRM production methods and their associated particle morphology, surface area, particle-size range, and binder effects are summarized in Table 5.

Grouping the routes in this way matters for the synthesis that follows. Only the first three rows change the particle itself; the surface treatments act on an existing particle, and terminal blending progressively removes the discrete particle altogether. Size-versus-property comparisons are therefore meaningful within a category but not across categories, and Section 5, Section 6 and Section 7 report them accordingly.

## 4. Swelling Kinetics and Interaction Mechanisms

The asphalt–rubber interaction mechanism was first clearly described by Abdelrahman and Carpenter [6], who identified particle swelling and degradation, including devulcanization and depolymerization, as the two major processes controlling the properties of CRM-modified binders. The interaction between crumb rubber and asphalt proceeds through a sequential physicochemical mechanism in which the rubber absorbs the lighter aromatic and resinous fractions (maltenes) of the binder, undergoes volumetric swelling, and under sufficient thermal energy gradually depolymerizes and dissolves into the asphalt matrix [42]. Earlier absorption-based work by Airey et al. [48], using the basket drainage method, confirmed that crumb rubber absorbs the lighter fractions of bitumen during interaction, producing residual binders with increased viscosity, complex modulus, and elastic response. Frantzis [49] further described bitumen uptake into rubber as a diffusion-controlled process approaching equilibrium, while Farouk et al. [50] reported greater bitumen absorption for finer rubber, consistent with its higher specific surface area. Ghavibazoo et al. [42] showed that interaction conditions, especially temperature and frequency, control the mechanism of CRM dissolution. Under lower interaction conditions, such as 160 °C and 10 Hz, CRM mainly absorbs aromatic components from asphalt and swells, whereas under severe interaction conditions, such as 220 °C and 50 Hz, CRM dissolves into the asphalt matrix and releases carbon black, fillers, polymeric components, and oily components. In a companion study, Ghavibazoo et al. [51] applied Stokes’ law to show that the storage stability of CRM-modified binders depends jointly on the reduction in particle size from dissolution and on the development of liquid-phase viscosity arising from released rubber polymers, establishing the link between the molecular-scale dissolution mechanism and the bulk-scale binder behavior. Peralta et al. [46] quantified the mutual chemical changes that occur in both phases during blending, confirming that the rubber and bitumen are jointly modified by the interaction rather than the rubber acting as an inert dispersed particle. Jeong et al. [14] showed experimentally that interaction time, interaction temperature, and rubber content significantly affect binder viscosity and high-temperature rheological properties, indicating that blending conditions control the extent of asphalt–rubber interaction. Consistent with earlier morphology findings, Shen et al. [11] showed that greater CRM surface area strengthens asphalt–rubber interaction and affects high-temperature rheological behavior.

**Figure 2:**
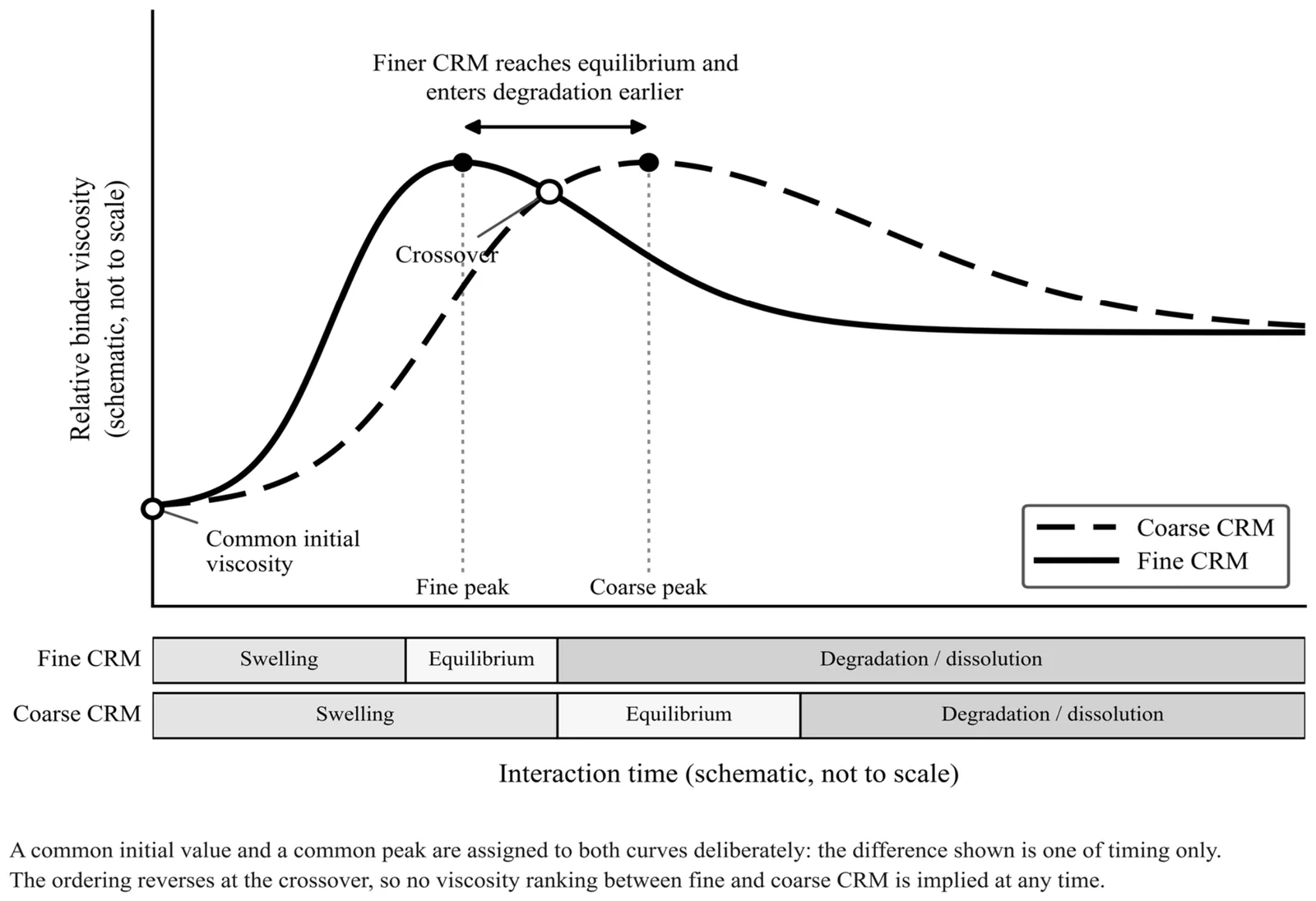
Schematic, not-to-scale representation of the viscosity-time evolution of fine and coarse CRM during the swelling, equilibrium, and degradation/dissolution stages [38,47,52]. Both curves are drawn from a common initial viscosity, because before interaction begins the two systems are the same base binder at the same CRM dosage, and to a common peak value, because reported peak magnitudes disagree in direction across studies. The only difference depicted is therefore one of timing: the earlier swelling equilibrium and earlier onset of degradation reported for finer CRM [38,47]. Because the two curves cross, neither particle size is drawn as giving a uniformly higher viscosity, and no viscosity ranking between fine and coarse CRM is implied at any time. The stage bands below the time axis are shown separately for each size because the stage boundaries differ in time rather than in magnitude.

Recent kinetic work shows that finer CRM reaches swelling equilibrium earlier and subsequently enters the degradation regime more rapidly because of its higher surface-area-to-volume ratio [38]. The extent of swelling and degradation also influences rheological and thermal behavior; during degradation at 200 °C, viscosity was reported to increase to a maximum of 20.58 Pa·s at 7.5 h before declining to 11.09 Pa·s after 35 h [52]. Santagata et al. [47] provided one of the most direct kinetic measurements available in the literature, reporting maximum swelling rates of 160 cP/min (0.16 Pa·s/min) at 22% CRM dosage and degradation rates of 55 cP/min (0.055 Pa·s/min), values that explicitly parameterize the rate processes for use in production optimization. Xing et al. [53] corroborated these kinetic trends within the bituminous matrix, showing that increasing treatment temperature and curing time can enhance crumb-rubber infiltration and modify viscosity and complex modulus; however, excessive treatment may cause serious rubber degradation and asphalt aging, leading to increased viscosity and stiffness. The conceptual viscosity-time evolution arising from these studies is illustrated in Figure 2, which highlights the swelling, equilibrium, and degradation regimes and the tendency of finer CRM to exhibit faster interaction kinetics.

Following Putman and Amirkhanian [7], the contribution of CRM to binder rheology can be decomposed into two co-existing mechanisms: particle effect and interaction effect. The particle effect (PE) describes the mechanical reinforcement provided by undissolved, swollen rubber particles acting as a dispersed elastic phase, whereas the interaction effect (IE) captures the rheological modification of the continuous asphalt phase arising from maltene absorption and partial rubber dissolution. Singh et al. [8] separated particle effect and interaction effect using undrained and drained CRM binders. In that study—one of the few to separate the two contributions experimentally—the particle effect was generally greater than the interaction effect for viscosity, G*/sinδ, percent recovery, and predicted binder fatigue life, while the interaction effect also contributed to nonrecoverable creep compliance. Their results showed that larger 30–40 mesh particles produced a stronger particle effect than smaller 60–80 mesh particles, whereas the smaller particles provided better high-temperature storage stability. Zheng et al. [54] extended this framework to the binder-aggregate interface, demonstrating that both effects influence interfacial adhesion: undissolved swollen particles provide physical reinforcement at the interface, while the interaction-modified continuous phase alters interfacial chemistry and moisture sensitivity. Ragab and Abdelrahman [55] connected the mechanism to bulk binder stability by showing that the interaction parameters (time, speed, temperature) directly control the development of a three-dimensional rubber-asphalt network whose interconnectivity determines storage stability; a more interconnected 3D network suppresses phase separation under hot storage. Wang et al. [56] formalized this network contribution within a micromechanics-based viscoelasticity model that explicitly incorporates polymer-chain entanglement and network effects, achieving substantially improved prediction of complex modulus and phase angle at low frequency and high temperature compared to conventional micromechanics models that omit interaction-derived network structure. At the multiscale level, Liu et al. [57] provided supporting evidence that CRM incorporation changes phase-transition behavior, molecular weight distribution, rheological response, adhesion, and nanomorphology, indicating that asphalt–rubber interaction modifies both the continuous binder phase and the dispersed rubber-modified microstructure. Collectively, these studies establish that swelling kinetics and interaction mechanisms are not independent phenomena; the rate and extent of swelling-degradation govern the balance between particle effect and interaction effect, and the resulting 3D network structure ultimately controls the bulk performance and storage stability of the modified binder.

## 5. Binder Response to Particle Size: High-Temperature Rheology (G*/sinδ, MSCR) and Intermediate-Temperature Fatigue (LAS)

**Figure 3:**
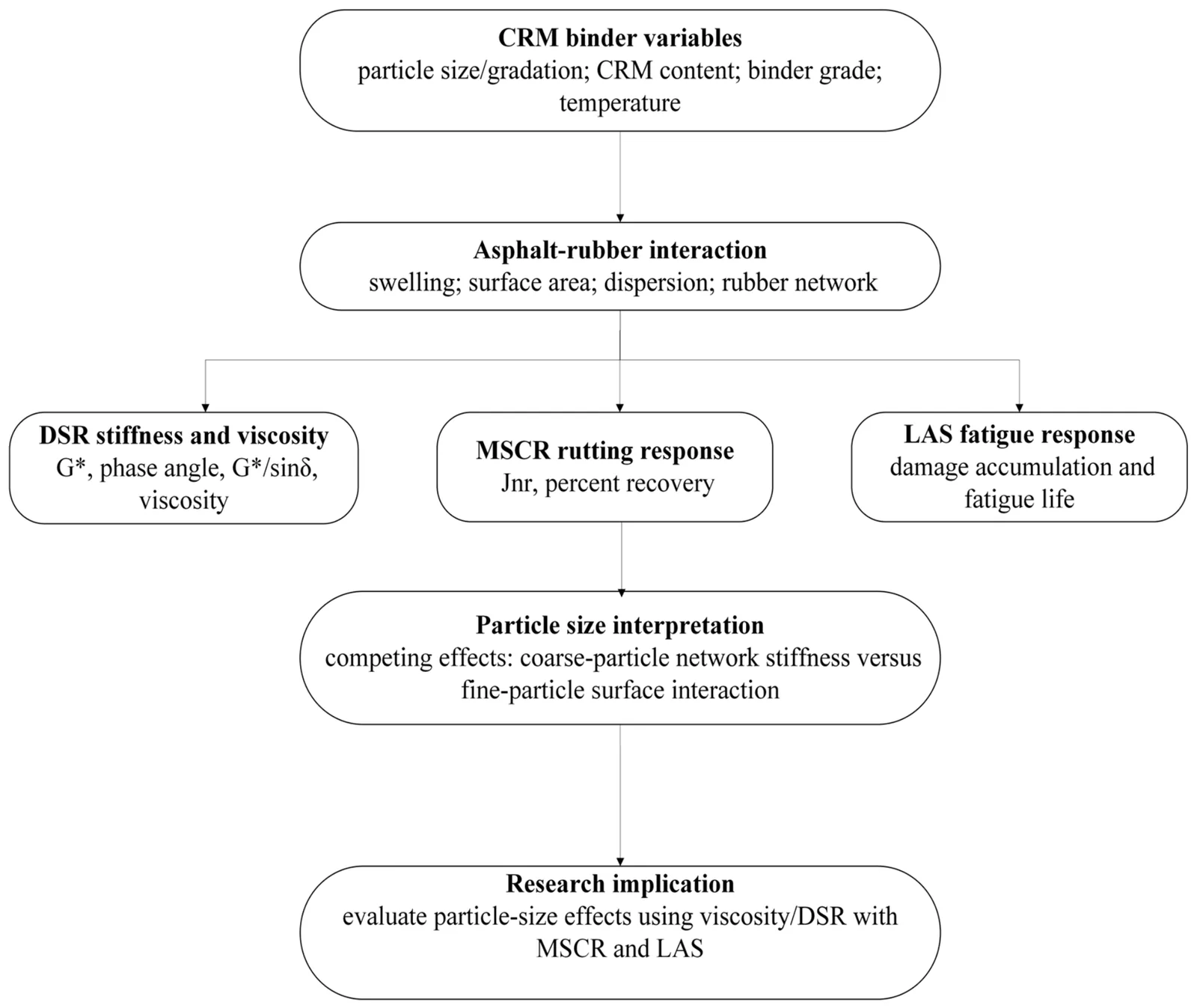
Schematic workflow linking CRM particle-size variables, asphalt–rubber interaction mechanisms, and binder-scale evaluation. DSR, dynamic shear rheometer; MSCR, multiple stress creep and recovery; LAS, linear amplitude sweep. The three tests evaluate different responses and do not form a hierarchy (Section 5). G* denotes the complex shear modulus; Jnr, non-recoverable creep compliance. Conceptual; authors’ synthesis.

The high-temperature rheological performance of CRM-modified binders governs their resistance to permanent deformation (rutting), while their fatigue performance governs resistance to load-associated cracking. These properties are evaluated using a hierarchy of dynamic shear rheometer (DSR) tests of increasing sophistication: the conventional Superpave rutting parameter G*/sinδ, the MSCR test [58], and the linear amplitude sweep (LAS) test. Crumb rubber particle size influences each of these responses by altering the volume fraction of swollen rubber, the stiffness of the dispersed rubber phase, and the degree of asphalt–rubber interaction in the continuous matrix [11,59]. This section reviews the reported effects of particle size on high-temperature rheology, MSCR response, and LAS fatigue, and highlights the contradictory findings that remain unresolved in the literature. The relationship between CRM particle size, asphalt–rubber interaction mechanisms, and rheological performance evaluation is summarized in Figure 3.

### 5.1. High-Temperature Stiffness and Viscosity

Early rheological work established that CRM raises the high-temperature complex and viscous moduli of the base binder. Navarro et al. [59] characterized ground tire rubber-modified bitumens across a range of particle sizes and found that rubber addition markedly increased the viscoelastic moduli and viscosity at low frequencies (high service temperatures), improving resistance to permanent deformation, with microstructural analysis attributing the increase to the larger, non-spherical rubber particles. Shen et al. [11] linked this stiffening directly to particle surface area, reporting that ambient CRM, with approximately twice the BET surface area of cryogenic CRM at equivalent gradation, produced a substantially higher complex modulus G* and a higher high-temperature grade. Because viscosity is sensitive to several interacting variables, Thodesen et al. [60] used statistical regression and neural-network models to predict CRM binder viscosity and found that asphalt binder grade, test temperature, and rubber content were the most important variables, whereas binder source and rubber gradation were relatively less influential. Xiao et al. [61] similarly showed that artificial-neural-network modeling could effectively predict CRM binder viscosity. At higher rubber dosages, Zhu et al. [62] and Qiao et al. [63] confirmed that increasing rubber content raises high-temperature stiffness, while Cai et al. [64] showed that increasing content shifts the particle size distribution toward finer and micro-particles as larger particles progressively degrade and dissolve during blending.

### 5.2. MSCR Response and the Limitations of G*/sinδ

The MSCR test, which reports the non-recoverable creep compliance (Jnr) and percent recovery (%R), provides a more sensitive high-temperature assessment for modified binders because it captures the delayed elastic response that the rubber network imparts. Tabatabaee and Tabatabaee [58] demonstrated that CRM-modified binders show an improved rutting-related binder response and higher elastic recovery under MSCR and reported good agreement between binder-level and mixture-level performance trends, supporting MSCR as a useful complementary tool for rutting evaluation. A recurring theme is that the conventional Superpave parameter G*/sinδ has limitations for CRM-modified binders. Bennert [19] found that MSCR with an increased DSR gap height correlated better with mixture rutting than conventional high-temperature performance grade (PG) grading. At the mixture scale, Xie et al. [65] compared candidate high-temperature indices for rubberized asphalt and recommended permanent deformation, softening point, and viscous stiffness modulus over rotational viscosity. More fundamentally, G*/sinδ has been shown to be inadequate for rating modified binders, which has prompted refinements of the parameter itself [66]. Zhang et al. [67] reinforced this conclusion, reporting that G*/sinδ failed to distinguish between different rubber particle sizes whereas MSCR best characterized high-temperature deformation. Warm-mix CRM studies further support this picture: Gui et al. [68] reported a positive synergistic effect of crumb rubber and warm-mix additives across several rutting indices, including the modified rutting factor G*/(sinδ)^9^, and Al-Khateeb et al. [69] reported improved rutting response and fatigue life with increasing rubber content, even when warm-mix additives are incorporated to mitigate the high viscosity of CRM binders.

### 5.3. The Particle Size Contradiction in High-Temperature Performance

The effect of particle size on high-temperature and rutting performance is genuinely contested in the literature, and a critical reading reveals two opposing positions. One body of work reports that coarser particles improve high-temperature performance: Navarro et al. [59] attributed higher low-frequency moduli to larger non-spherical particles; Qian et al. [70] found that larger crumb-rubber particles improved rutting and fatigue resistance in CRM composite binders while finer particles favored low-temperature cracking resistance and storage stability. Lei et al. [12] have been read as belonging to this group as well, because the abstract of that paper describes crumb rubber size as positively correlated with fatigue life. The body of that paper does not support the reading: predicted fatigue life there rose with increasing mesh number; that is, with decreasing particle size. The finest fraction tested, 100 mesh (0.15 mm), gave both the longest predicted fatigue life and the highest crack-propagation parameter. The shorter lives of the coarser blends are attributed to crack propagation in the vicinity of incompletely depolymerized rubber. That study therefore favors the finer fractions for fatigue, while its optimum for storage stability was a mid-range 40 mesh, with 80 mesh close to it. A size effect on the high-temperature elastic response was not reported in that study. A second body of work reports that finer or well-dispersed rubber fractions can improve high-temperature performance under certain conditions. Wang et al. [9] reported that finer crumb rubber attained higher high-temperature viscosity than coarser rubber. Guo et al. [71] further found that finer CRM fractions in CRM-modified binders produced more significant improvements in high and low-temperature performance, although the role of coarser particles became more prominent after aging. Cai et al. [64] showed that medium and small rubber-particle fractions can reduce non-recoverable creep compliance and improve fatigue resistance, whereas larger particles mainly enhance high-temperature deformation resistance. This contradiction is best understood as arising from differences in base binder, rubber content, production method, and critically the competing mechanisms identified: coarser particles contribute a stronger particle (physical reinforcement) effect, whereas finer particles, with their higher surface area, contribute a stronger interaction effect and reach more complete swelling. The net rheological outcome depends on which mechanism dominates under the specific blending and testing conditions employed, which explains why no single particle size emerges as universally optimal [15,72]. The contrasting particle-size effects reported across the reviewed studies are summarized in Table 6.

### 5.4. Fatigue Response (LAS and Time Sweep)

Fatigue resistance, assessed by the LAS test and validated against time-sweep testing, is consistently improved by crumb rubber, though the particle size dependence is again nuanced. Nan et al. [73] showed that crumb rubber significantly improved fatigue life relative to the base binder, with the CR composite binder showing the strongest fatigue performance; LAS and time-sweep fatigue rankings were also strongly correlated. Surface treatment of the rubber further enhances fatigue performance: Li et al. [74] demonstrated that low-temperature plasma treatment of the rubber surface improved both LAS fatigue performance and storage stability, while producing only a slight change in low-temperature performance. Particle size and gradation enter the fatigue picture through several routes: Zhang et al. [67] evaluated rubber-modified asphalt using temperature sweep, MSCR, time-sweep, LAS, bending beam rheometer (BBR), and differential scanning calorimetry (DSC) tests and reported that asphalt containing 40-mesh rubber exhibited the optimum overall rheological behavior. Complementary investigations of aging and high-cured systems by Wang et al. [75] and the broader synthesis of Duan et al. [24] indicate that the rubber network continues to govern fatigue and high-temperature behavior throughout the binder service life, with the balance between physical reinforcement and rubber-asphalt interaction evolving as the binder ages (an interpretation, not a directly measured partition). Venudharan et al. [76] separately report that crumb rubber influences the chemical and thermal properties of the binder as well as its physical characteristics, and that higher dosages combined with finer gradations produced the best-performing asphalt–rubber binders.

## 6. Storage Stability and Phase Separation

Wet-process CRM-modified binders are inherently two-phase systems in which swollen, partially dissolved rubber particles are dispersed in a continuous asphalt matrix. When such binders are stored or transported at elevated temperatures without agitation, the dispersed rubber tends to separate from the matrix, producing a vertical gradient in composition and rheological properties. The practical consequences are severe: settling rubber can clog the drain valves of storage-tank pumps, so unstable binders must be held in continuously stirred tanks at additional energy and equipment cost [77]. This phase separation is widely regarded as one of the principal obstacles to the wider use of CRM-modified binders, particularly for terminal-blend products that must remain homogeneous between the blending terminal and the paving site [41]. Because particle settling is strongly size-dependent, storage stability is closely linked to CRM particle size.

### 6.1. Mechanisms of Phase Separation

CRM swelling, degradation, and interaction-state evolution modify both the dispersed rubber and continuous asphalt phases. For storage stability, the key consequence is the development of a stabilizing network and changes in residual particle size and matrix viscosity.

**Figure 4:**
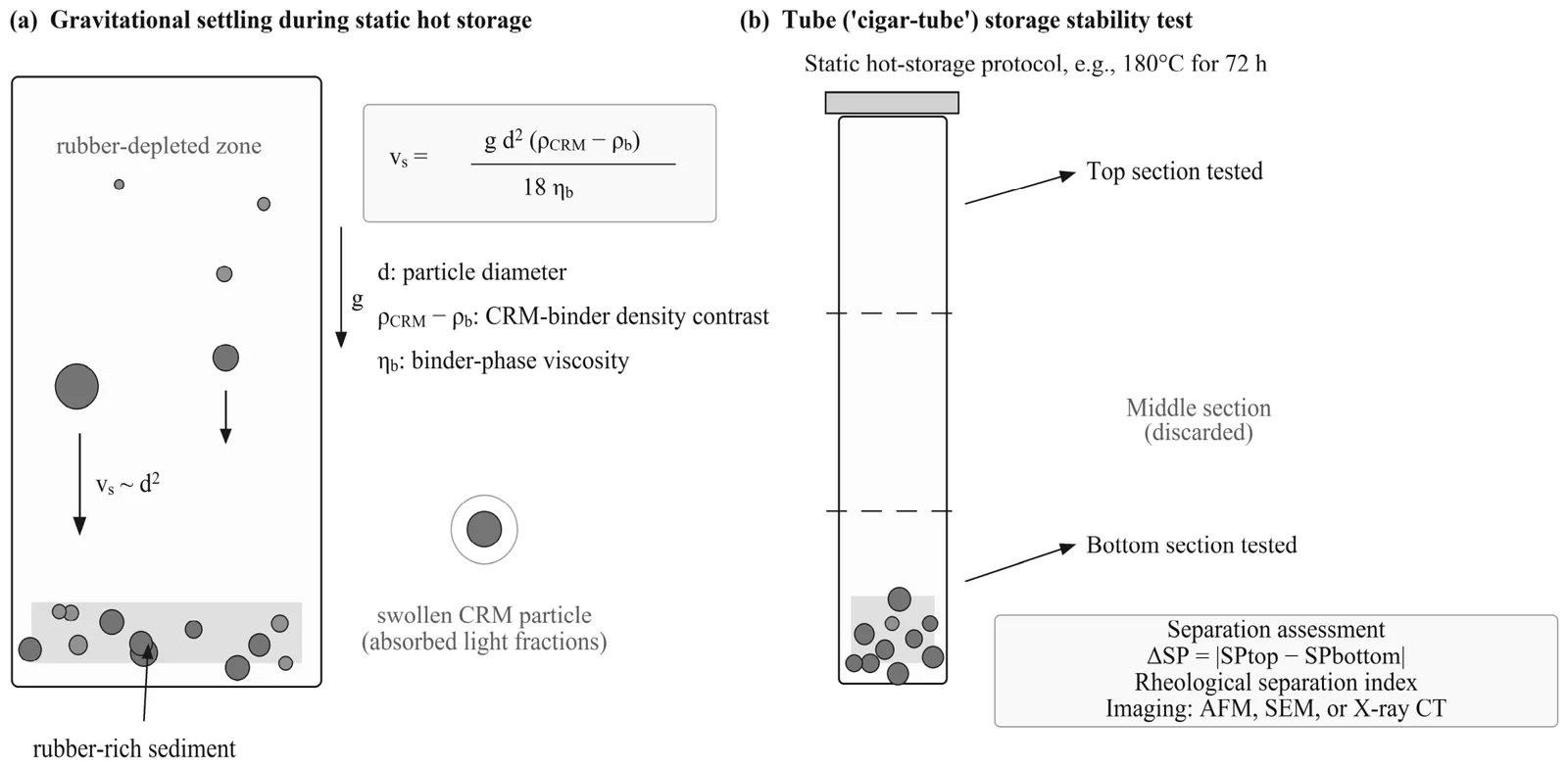
Schematic of gravitational settling and tube-test evaluation of CRM binder storage stability, after the procedures described in [13,51,79]. SI, separation index; ΔSP, softening-point difference. The settling schematic illustrates the Stokes-law reasoning of Section 6.1 and its stated limitations; it is not to scale and predicts no settling rate. The open ring drawn around the particle in panel (**a**) denotes the layer of light fractions absorbed by a swollen CRM particle. AFM, atomic force microscopy; SEM, scanning electron microscopy; CT, computed tomography. The density and viscosity symbols in panel (**a**) are those of the CRM particle and of the binder phase; they correspond to ρp, ρf and η in the equation in Section 6.1.

Where there is no stabilizing structure, the process resembles gravitational settling of high-density particles in a viscous liquid. Accordingly, some studies have interpreted this behavior using Stokes’ equation [51,77] (Figure 4):

v = [d^2^ (ρ_p_ − ρ_f_) g]/(18 η)

where v is the terminal settling velocity of a rubber particle (m s^−1^), d the particle diameter (m), ρ_p_ and ρ_f_ the densities of the rubber particle and of the liquid phase (kg m^−3^), g the gravitational acceleration (9.81 m s^−2^), and η the dynamic viscosity of the liquid phase (Pa·s). The quadratic dependence on d is the reason particle size dominates settling behavior in the absence of a stabilizing network.

Four of the assumptions behind this expression are not satisfied by CRM binders, and the equation is therefore used here only as a qualitative framework, never as a predictor of settling velocity. (i) Stokes’ law assumes rigid spheres, whereas CRM particles are irregular, deformable, and swollen. (ii) It assumes a Newtonian continuous phase, whereas the asphalt matrix is strongly non-Newtonian and shear-thinning at storage temperature. (iii) It assumes constant d, ρ_p_ and η, whereas all three evolve during storage as swelling and dissolution proceed. (iv) It assumes dilute, non-interacting particles, whereas CRM dosages of 10–22% by binder mass place the system well into the hindered-settling regime. The expression is retained in this review because it identifies the three levers that the experimental literature confirms—residual particle size, density contrast, and liquid-phase viscosity—not because it quantifies them. Based on this proportionality, Sienkiewicz et al. [77] identified three approaches for achieving a stable CRM-modified binder: reducing the particle size of the rubber, reducing the density difference between the rubber and bitumen, and increasing the viscosity of the liquid phase. In line with this idea, rubber tends to concentrate in the lower portion of stored samples: Yun et al. [78] reported improved rheological characteristics in the lower portions of CRM binders, relating it to the different densities of rubber and base asphalt, whereas Liang et al. [43] observed corresponding top-bottom differences in frequency-sweep viscoelastic response. From the Stokes-law perspective, asphalt–rubber interaction is as important as the initial rubber particle size because interaction can reduce the residual particle size and increase the viscosity of the liquid phase, both of which suppress particle settling. For example, Ghavibazoo et al. [51] demonstrated that, at an interaction temperature of 190 °C, dissolution improves storage stability through two simultaneous mechanisms: a decrease in residual particle size and an increase in liquid-phase viscosity. However, increasing the interaction temperature to 220 °C alters the dissolution mechanism and the nature of the residual particles.

### 6.2. Effect of Crumb Rubber Particle Size

Navarro et al. [10] prepared 60/70 penetration-grade bitumen modified with ground tire rubber particles having average sizes ranging from 0.10 to 0.74 mm. They reported that storage stability decreased as particle size increased; however, the ≈0.29 mm fraction remained stable after 12 h of hot storage at 150–180 °C, whereas the larger fractions were unstable [10,77]. It was concluded that a CRM fraction smaller than 0.35 mm should be used together with high-shear mixing. Cao et al. [80] evaluated five crumb-rubber particle sizes, 20, 40, 60, 80, and 120 mesh, at three rubber contents of 10%, 15%, and 20%. They found that particle size and rubber content significantly affected conventional binder properties, including penetration, softening point, ductility, and viscosity, and identified 80-mesh rubber at 15% content as the optimum combination based on performance and field workability. Subsequent experimental studies broadly confirm this inverse relationship between CRM particle size and storage stability. Across commercial CRM products and controlled particle-size comparisons, finer CRM generally produced less phase separation and more homogeneous top-bottom microstructures, while recent X-ray computed tomography (CT) measurements reported strong correlations between particle size and storage stability (R^2^ > 0.75 under all tested storage conditions) [13,36,78,79]. However, Vigneswaran et al. [13] also demonstrated the associated performance trade-off: CRM ≤ 0.5 mm provided the greatest storage stability, whereas 1–2 mm CRM gave a more favorable rutting-related binder response and higher percent recovery but poorer workability.

Particle-size effects remain relevant in chemically modified and composite binders, although the trends are more nuanced. For example, Hosseinnezhad et al. [34] showed that surface functionalization of crumb rubber through microwave-assisted bio-modification substantially reduced phase separation and improved rubber-asphalt compatibility. In CRM-modified binders, Qian et al. [70] showed that small rubber particles enhanced storage stability and low-temperature crack resistance, while large particles were more favorable for rutting and fatigue resistance. Importantly, the optimum particle size is not always the smallest: in bio-oil pre-treated composite binders, Lei et al. [12] selected 40 mesh, a mid-range size, as optimal for storage stability among 20, 40, 80 and 100 mesh rubbers, whereas within the same study the finest fraction gave the longest predicted fatigue life, so that the two properties did not select the same size. Together with the 80-mesh optimum proposed by Cao et al. [80], the above results demonstrate that particle-size selection for storage stability is an optimization against other performance requirements rather than a straightforward minimization.

Two limitations affect the relationship between particle size and storage stability. First, particle size is not independent of processing because swelling and dissolution change the residual particle size [51]. Storage stability is also influenced by rubber content and source [41], and higher rubber contents can improve stability, potentially through network formation and increased viscosity [81]. Second, initial particle size does not necessarily control the dissolution rate. Medina et al. [32] reported no effect of rubber size (125–250 μm versus 125–800 μm) on the dissolution rate under their production conditions. Microstructural studies nevertheless indicate that finer particles gradually become less distinguishable within the asphalt matrix, suggesting improved rubber-asphalt compatibility [43]. Table 7 presents the main experimental research on the link between size and storage stability.

Two limits of this compilation should be read alongside it. First, the sizes in the third column are the sizes charged to the blend; the size that governs settling is the residual size after swelling and dissolution, which only Ghavibazoo et al. [51] resolve explicitly. Second, the three index families in the second column measure different things—an empirical softening- point difference, a rheological ratio between tube sections, and a tomographic volume fraction—and Cao et al. [79], who measured both on the same materials, report a significant but incomplete agreement between the tomographic indices and ΔSP, with correlation coefficients near 0.8; the tomographic indices were validated against ΔSP rather than shown to supersede it. Comparisons are therefore made within an index family and not across families.

### 6.3. Assessment of Storage Stability

The procedure for storage stability still relies on the hot-storage tube test (cigar-tube method), during which the sample is kept in the upright position in a vertical tube at a high temperature, and then the top and bottom sections of the sample are tested (Figure 4b). As summarized by Sienkiewicz et al. [77], EN 13399:2010 [84] specifies that modified bitumen samples are stored vertically at 180 °C for 72 h, cooled, and divided into sections, after which storage stability is assessed by comparing the penetration and softening-point values of the upper and lower sections. However, storage-stability conditions vary across studies, with reported storage times ranging from 12 to 240 h and temperatures ranging from 140 to 200 °C [77]. The conventional assessment commonly relies on the difference between the softening-point values of the upper and lower sections of the stored sample. Because a single empirical softening point difference may not fully capture viscoelastic separation, rheological separation indices are now commonly used, including G*/sinδ ratio and percent recovery comparison between top and bottom sections [13], frequency-sweep comparison of top and bottom layers [43], as well as separation indices built from differences in mechanical property and rubber content between tube sections [81]. Alternative approaches include microstructural evaluation: atomic force microscopy (AFM) imaging of top and bottom layers [78], scanning electron microscopy (SEM) observation of rubber-particle distribution [30], and more quantitatively, X-ray CT-based separation indices in terms of area and volume fraction, which Cao et al. [79] reported to be more reliable than the traditional softening point difference. In their review of separation techniques for terminal-blend rubberized binders, Wen et al. [41] concluded that measured storage stability depends jointly on rubber content, rubber source, and particle size.

### 6.4. Influence of Processing and Hot-Storage Conditions

Storage stability is influenced not only by the presence of additional agents but also by the processing and storage conditions of the binder. Interaction parameters, such as time, speed, and temperature of the process, affect the formation of the stabilizing inner structural network [55] and, as mentioned above, the temperature of interaction changes the separation mechanism [51]. Overprocessing can be detrimental. Cao et al. [80] reported a sharp decline in viscosity when the reaction temperature exceeded 200 °C or the reaction time exceeded 60 min, with further performance deterioration after 4 h of high-temperature processing. Processing and subsequent hot-storage conditions strongly affect CRM-binder evolution, with the response depending on rubber production method, interaction temperature, and storage temperature. Continuous viscosity measurements showed that processing at 195 °C produced peak viscosity after approximately 45–60 min, while subsequent storage at 150 °C minimized viscosity loss. Prolonged low-shear storage at 180 °C also affected ambient and cryogenic-ground CRM differently, demonstrating that the effects of hot storage are material dependent rather than universally consistent [30,32]. Storage temperature also interacts with chemical stabilization, and Qiao et al. [63] accordingly evaluated the stability of high-content rubber CRM-modified asphalt at different storage temperatures using Fourier-transform infrared spectroscopy (FTIR), DSC and rheological tests. Finally, the storage stage constrains formulation choices: based on rheological and X-ray CT-derived separation indices, Wang et al. [81] advise against introducing warm-mix additives into CRM bitumen during the storage or transport stage.

### 6.5. Strategies for Improving Storage Stability

**Figure 5:**
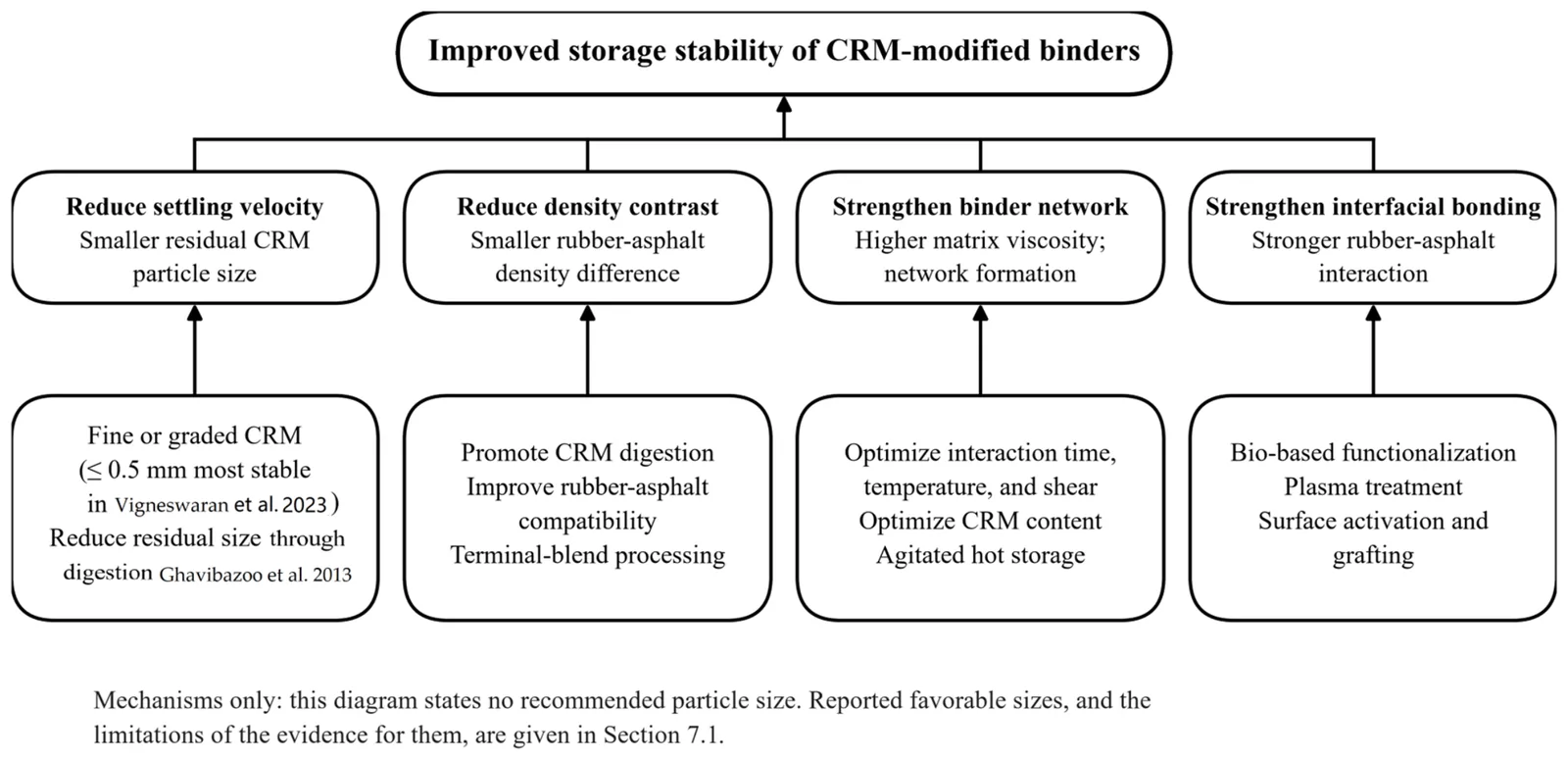
Synthesis of strategies reported to improve the storage stability of CRM-modified binders, grouped by the physical mechanism each targets, following the three mechanistic routes of Yang et al. [25] mapped onto the qualitative Stokes variables of Section 6.1 [12,13,23,30,34,51,55,63,74,77,85]. The diagram represents mechanisms only and states no recommended particle size; reported favorable sizes, and the limitations of the evidence for them, are given in Section 7.1. The size shown against Ref. [13] is the result reported by that single study, not a design recommendation.

Reviewing the improvement methods reported to date, Yang et al. [25] group them into three mechanistic routes: increasing the swelling rate of the rubber, promoting crosslinking between rubber and bitumen, and reducing rubber agglomeration. Most reported strategies can accordingly be read as acting on one of the variables of the Stokes settling problem particle size, density contrast or matrix viscosity [77] or as replacing purely physical dispersion with interfacial bonding (Figure 5).

Surface treatments can improve storage stability by increasing CRM surface activity and strengthening rubber–asphalt interaction. Bio-based functionalization and plasma treatment have been shown to reduce phase separation and improve compatibility, while activation and grafting approaches similarly promote stronger interfacial interaction [23,34,74,77]. These findings indicate that surface modification can partly compensate for limitations associated with particle size by increasing the effective interaction area and interfacial bonding.

Storage stability can also be improved by controlling the other mechanisms summarized in Figure 5. Chemical coupling and pretreatments can strengthen the rubber–asphalt network or promote swelling and digestion, while reducing the density contrast between the dispersed and continuous phases provides another means of suppressing separation [12,63,77,85]. Appropriate interaction time, temperature, shear, and rubber content can further promote dispersion and development of a stabilizing network [55,62]; prolonged agitated hot storage, however, is not universally beneficial, having been reported to be significantly detrimental for ambient-ground CRM while leaving cryogenically ground CRM largely unaffected [30]. At the limiting case, terminal-blend processing produces a more homogeneous and storage-stable binder through extensive digestion of fine CRM, although some discrete-particle reinforcement may be lost [77].

## 7. Discussion, Recommendations, and Future Standardization

### 7.1. What the Evidence Supports on Crumb Rubber Size Selection

The evidence reviewed for high-temperature rheology and storage stability does not identify a single generally applicable optimum. Instead, several study-specific favorable sizes fall within a fine-to-intermediate region spanning approximately 40–80 mesh. These values were selected using different criteria: overall binder performance [80], storage stability [10,12,13], and overall rheological behavior [67]. The interval therefore describes where criterion-specific findings happen to cluster; it is not optimized against any single criterion and should not be interpreted as a size that is simultaneously best for every property. It is neither statistically derived nor universally applicable. Rubber content also strongly influences modification and cannot be selected independently of particle size [16]. Because reported dosages vary widely and dosage optimization was outside the scope of this particle-size-focused review, no single CRM content range is recommended. Substantial differences in base binder, rubber source, grinding method, CRM content, and processing conditions further limit transferability. The observed cluster should therefore not be treated as a design recommendation or specification limit. Particle size remains a design variable that must be considered jointly with rubber content and processing conditions. The overall effects of decreasing CRM particle size on binder properties and related performance indicators are summarized in Table 8.

Low-temperature cracking resistance and asphalt-aggregate adhesion were reported in an earlier version of this table. They have been removed because neither is developed in the body of this review, and retaining them in a summary table implied a synthesis that was not performed. Both remain open questions for a size-organized review of low-temperature and interfacial behavior.

To make the basis of the provisional evidence cluster explicit, the study-specific favorable sizes are compiled on a common metric basis in Table 9.

Of the six studies in Table 9, three report a favorable size that falls within the 0.180–0.425 mm interval on a comparable retained-fraction basis [12,67,80]; one reports an upper bound that overlaps the interval without being contained by it [10]; one reports a favorable fraction that extends to a coarser range than the interval [13]; and one reports no optimum at all [37]. Two of the three studies inside the interval sit exactly on its bounds. The interval is therefore better described as the region in which a small number of heterogeneous, criterion-specific optima happen to cluster than as a design window.

### 7.2. Research and Testing Needs for Future Standardization

Existing binder-characterization frameworks are not fully suited to particulate CRM binders because coarse and deformable rubber particles can interfere with conventional test geometries. MSCR-based studies indicate that particle-compatible test configurations, including appropriate DSR gap heights, provide better discrimination of CRM behavior than conventional high-temperature PG parameters [19,67]. In one study that compared particle sizes directly, G*/sinδ did not distinguish between rubber particle sizes whereas MSCR parameters did [67]. Independent replication of this comparison is needed before it can be treated as an established property of the two parameters. Binder-level results should nevertheless be validated against mixture-level performance [58].

Greater consistency in routine quality-control measurements is needed before particle-size-specific testing procedures can be standardized. Ding et al. [20] demonstrated that CRM binder viscosity measurements are strongly affected by rotor model, rotational speed, torque range, and rubber content, and they recommended appropriate rotor-speed combinations with torque-based interpolation to improve the effectiveness and comparability of viscosity testing. The need for such corrections in a fundamental measurement such as rotational viscosity demonstrates the substantial influence of particle characteristics on binder characterization. Without consistent testing conditions, variations caused by equipment configuration or operating parameters may be incorrectly interpreted as differences in material behavior.

### 7.3. Predictive Frameworks and Future Research Directions

One promising approach to addressing these testing and characterization limitations is to predict CRM-binder performance from rubber gradation. Venudharan and Biligiri [87] showed that crumb-rubber gradation can be used for rheological evaluation, optimization, and selection of asphalt binders, supporting the use of particle gradation as a design variable rather than only a descriptive material property. Venudharan and Biligiri [88] further demonstrated that gradation, binder properties, and test parameters can be used to estimate rutting-performance indicators such as viscosity, G*/sinδ and tanδ. Together, these studies shift the emphasis from prescribing isolated particle-size limits toward treating gradation as a design variable within a performance-based specification framework.

**Figure 6:**
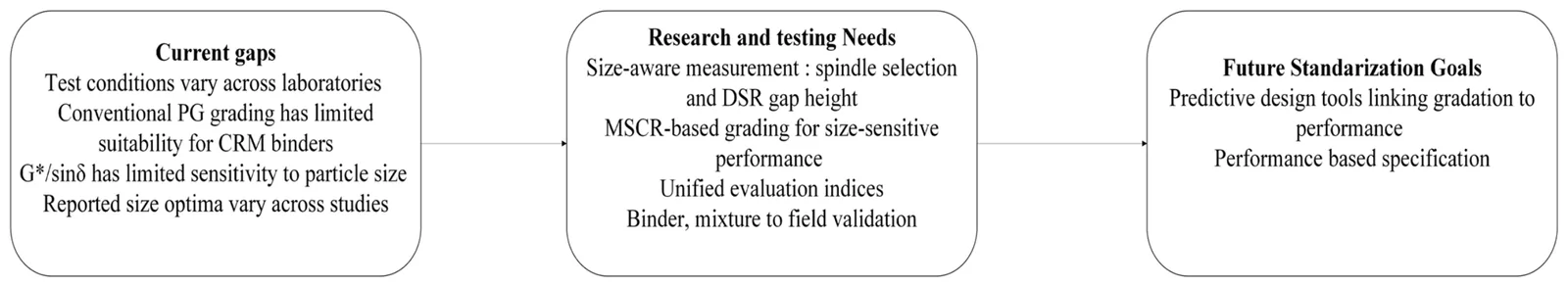
Roadmap linking the research gaps identified in Section 7 to the testing and standardization work they imply. The figure is the authors’ synthesis of the gaps stated in the text and does not itself report evidence. G* denotes the complex shear modulus; PG, performance grade; DSR, dynamic shear rheometer; MSCR, multiple stress creep and recovery.

In summary, these review findings indicate five key considerations for future research. First, there is a need to develop and validate consistent particle-size-aware testing protocols, including the protocols for spindle selection and gap height for dynamic shear rheometer tests in particular, and for size-specific grading procedures, following the rotor and rotational-speed recommendations of Ding et al. [20] and the size-based grading approach developed by Bennert [19]. Second, it is recommended to develop a consistent set of evaluation criteria based on the MSCR-type indices identified by Zhang et al. [67] as sensitive to size, rather than on the insensitive traditional rutting factor. Third, binder-level findings should be validated against mixture-scale performance, as recommended by Tabatabaee and Tabatabaee [58], and, beyond that, against field performance. Fourth, there is a need for performance-based specification of particle size using size-related performance models, such as that developed by Venudharan and Biligiri [88]. Fifth, a unified storage-stability metric is needed to enable reliable comparison across particle sizes and processing conditions, preferably integrating rheological indices with quantitative imaging-based assessment. These research priorities and their relationship to particle-size-aware standardization are summarized in Figure 6.

### 7.4. Limitations of Available Evidence and the Present Review

Interpretation of crumb-rubber particle-size effects is constrained by substantial methodological variation among the reviewed studies. Particle size is reported using mesh designation, maximum size, mean diameter, or broad gradation ranges, which are not always directly comparable. Moreover, nominally similar particle sizes may differ in morphology, surface area, tire source, and grinding method. Particle-size effects are also frequently confounded by variations in rubber content, base-binder grade, blending temperature, interaction time, shear conditions, and the use of pretreatments or additional modifiers. Consequently, some differences attributed to particle size may partly reflect variations in material composition or processing conditions.

Comparison of rheological and storage-stability results is further limited by differences in test geometry, DSR gap height, rotational-viscosity spindle and speed, storage temperature and duration, and the indices used to quantify phase separation. Coarse CRM particles may also interfere with conventional binder-test geometries, reducing the direct comparability of reported results. In addition, much of the available evidence is derived from binder-scale laboratory testing, with comparatively limited validation at the mixture and field scales. Because the reviewed studies differed substantially in materials, processing conditions, particle-size definitions, and performance measures, the findings were synthesized qualitatively rather than combined statistically. In addition, because the literature search was restricted to English-language publications, potentially relevant evidence published in other languages may not have been captured, introducing a possible language-selection limitation. Accordingly, the particle-size interval described in this review should be read as a provisional evidence cluster rather than as design guidance or a universal specification limit.

## 8. Conclusions

This review examined crumb-rubber particle size as a primary factor governing the behavior of CRM-modified asphalt binders. Based on these studies, the main conclusions are:

1. Particle size is a controlling design variable. Its effect can be interpreted through the balance between the particle effect, in which coarser swollen rubber provides elastic reinforcement, and the interaction effect, in which finer particles with greater surface area promote maltene absorption and modification of the continuous asphalt phase.
2. This balance explains much of the apparent disagreement in the literature. Coarser particles may improve high-temperature response through stronger physical reinforcement, whereas finer particles can enhance binder interaction. The dominant response depends on the base binder, rubber content, particle size, and processing conditions.
3. The conventional G*/sinδ parameter appears to have limited sensitivity to particle-size-dependent behavior in CRM binders. Direct evidence for this specific limitation currently rests on a single comparative study [67]; the wider case against G*/sinδ for rubber-modified binders rests on separate grounds, namely test-geometry effects [19] and the demonstrated inadequacy of the parameter for rating modified binders [66]. On present evidence MSCR provides a more suitable assessment of high-temperature deformation, while LAS offers complementary evaluation of fatigue performance, but the particle-size discrimination of MSCR would benefit from independent confirmation.
4. The synthesis identifies approximately 40–80 mesh (0.180–0.425 mm, ASTM E11) as a provisional evidence cluster within which several study-specific favorable responses have been reported. Of the six studies compiled in Table 9, three fall inside the interval on a comparable basis, one overlaps it, one extends coarser, and one reports no optimum. The interval should be read as a description of where the present evidence happens to concentrate, not as a design window or specification limit. This range is not a universal optimum and should be co-optimized with rubber content, binder characteristics, and processing conditions.
5. Future efforts toward size-aware specifications should consider improved testing and evaluation procedures. These include particle-size-sensitive test protocols, appropriate DSR gaps and viscosity-testing conditions, MSCR-based criteria, unified storage-stability assessment, and validation at mixture and field scales.

Overall, incorporating CRM particle size into performance-based testing and specifications is an important step toward more reliable and standardized use of CRM-modified asphalt binders.

## Figures and Tables

**Table 1:** Scope of previous reviews of CRM-modified binders and the contribution of the present review. Entries are taken from the stated scope of each review; where a review’s abstract could not be obtained, this is noted rather than inferred. Arrows denote the sequence of the causal chain, not a quantitative relationship. CRM, crumb rubber modifier; MSCR, multiple stress creep and recovery.

Review	Primary Focus	Period/Basis	Organizing Variable	Treatment of Particle Size	Output
Lo Presti [5]	Recycled-tire-rubber modified bitumens: technologies, production, handling, storage, applications	Narrative, to 2013	Technology and process	Mentioned among fabrication parameters	State-of-practice overview
Ibrahim et al. [4]	Effect of crumb rubber addition on binder rheology	Narrative	Rheological property	Dedicated treatment as one internal factor among quantity, base-bitumen composition, mixing time and temperature	Narrative synthesis of rheological effects
Bressi et al. [21]	State of the art plus statistics on fabrication parameters and binder properties	Statistical over a sampled publication set, to 2019	Fabrication parameter	Quantified statistically (distribution of reported sizes across the sampled literature)	Descriptive statistics of practice
Zheng et al. [23]	Compatibility between crumb rubber and asphalt binder	Narrative	Compatibility	Discussed as one factor influencing compatibility	Evaluation methods and improvement approaches
Yang et al. [25]	Methods for improving storage stability of rubber bitumen	Narrative (abstract not obtained; scope taken from title)	Stabilization method	Not established from the available record	Grouping of stabilization routes
Lyu et al. [26]	Aging evolution and sustainability implications	Systematic literature review, 1993–2022	Aging	Not the organizing variable	Aging research map and life-cycle findings
Present review	Particle size across the full causal chain: production route → morphology and surface area → swelling and interaction → rheology, fatigue response, storage stability → specification needs	Structured narrative synthesis, searches May 2026, 83 included sources	Particle size	The organizing variable throughout	(i) particle-effect/interaction-effect reconciliation of conflicting findings; (ii) criterion-by-criterion compilation of favorable sizes on a common ASTM E11 [27] metric basis (Section 7.1); (iii) an explicit statement of what the evidence does not support, including the MSCR sensitivity hypothesis

**Table 2:** Search strategy by database. The strategy was first executed in May 2026 and re-executed so that the yield can be verified independently. Each database records its own re-execution date; the Transport Research International Documentation (TRID) database is openly searchable, whereas Scopus and Web of Science require a subscription. The total is the combined yield of the three searches before de-duplication and is not a stage-by-stage screening count (Section 2.4).

Database	Platform/Segment	Fields Searched	Records Returned on Re-Execution
Scopus	Elsevier	Title, abstract, keywords	511 (re-executed 9 September 2026)
Web of Science	Core Collection	Topic	299 (re-executed 9 September 2026)
TRID	TRB/OECD ITRD	All fields	195 (re-executed 8 September 2026)
Citation tracking and hand-searching	—	—	not applicable
Total	—	—	1005 records before de-duplication

**Table 3:** Inclusion and exclusion criteria, with the rationale applied during screening.

Criterion	Included	Excluded	Rationale
Scale of evidence	Binder-scale measurement on CRM-modified binder	Studies reporting only mixture or pavement outcomes	The review question concerns the binder phase
Size reporting	CRM particle size, size fraction, or gradation reported	Size not reported or described only as “crumb rubber”	Size is the organizing variable; unreported size cannot be placed on the size axis
Outcome	asphalt–rubber interaction, rheological response, fatigue response, or storage stability	Outcomes unrelated to these four domains	Defines the synthesis scope
Language	English	Other languages	Screening capacity of the review team
Publication type	Peer-reviewed articles; authoritative agency reports and standards	Theses, abstracts, non-peer-reviewed items	Quality floor

**Table 4:** ASTM E11 sieve designations and nominal openings used in this review.

Mesh (ASTM E11)	Nominal Opening (mm)	Mesh (ASTM E11)	Nominal Opening (mm)
No. 10	2.00	No. 60	0.250
No. 16	1.18	No. 80	0.180
No. 20	0.850	No. 100	0.150
No. 30	0.600	No. 120	0.125
No. 40	0.425	No. 200	0.075
No. 50	0.300		

**Table 5:** CRM production and incorporation routes, grouped by the type of operation they perform. Size-reduction routes determine the particle produced; post-treatments modify the particle surface without substantially changing bulk size; incorporation routes determine the state of the rubber in the binder. Mesh values are ASTM E11 (Table 4). BET denotes the Brunauer–Emmett–Teller gas-adsorption method for specific surface area. G* denotes the complex shear modulus.

Category	Route	Morphology	Specific Surface Area	Particle Size	Key Binder Effect	Refs.
Size reduction	Ambient grinding	Rough, fibrous, porous	Higher than cryogenic at matched size; across commercial products the route effect is confounded by size	0.5–5 mm; down to 40 mesh (0.425 mm)	Higher viscosity and G* than cryogenic-ground CRM at matched size; MSCR response and storage stability varied with study conditions	[11,15,29,32,36]
Size reduction	Cryogenic grinding	Smooth, glassy, planar	Low (≈ ½ ambient)	30–300 mesh (≈0.60–0.05 mm)	Lower viscosity and improved workability relative to ambient-ground CRM	[15,29,30]
Size reduction	Water-jet cutting	Rough, high asperity	Higher than mechanical shredding or cryogenic	Comparable to ambient-ground CRM	Favorable low-temperature binder response relative to the comparison CRM in Ref. [28]	[28]
Post-treatment	Devulcanization	Micro- to nano-scale, altered chemistry	Very high	<50 µm	Uniform dispersion, high compatibility	[33]
Post-treatment	Surface activation	Grafted biomolecules [34]; mainly physical change [35]	Increased vs. untreated	Bulk size essentially unchanged	Reduced separation and improved workability relative to untreated CRM [34]; modestly improved high-temperature binder response relative to untreated CRM [35]	[34,35]
Incorporation route	Terminal blend	Progressive dissolution	Not applicable after dissolution	Fine CRM preferred	Homogeneous, storage-stable binder	[40,41,46]

**Table 6:** Conflict matrix of reported particle-size effects on binder response. The final column states whether the study varied particle size as an isolated experimental factor or whether the size effect was inferred from a comparison in which other variables also differed. Blending, aging, and test conditions are given as reported in the source; “not reported” indicates the source does not state the value. SBS, styrene-butadiene-styrene.

Study	Particle-Size Comparison	Property Considered	Reported Size Effect	Interpretation	Size Isolated?
Navarro et al. [59]	Larger vs. smaller CRM particles	High-temperature viscoelastic response	Coarser favored	Larger non-spherical particles increased low-frequency moduli, indicating stronger particle reinforcement	Isolated
Qian et al. [70]	Larger vs. smaller CRM particles, with SBS co-modification	Rutting-related response, fatigue, low-temperature cracking, storage stability	Property-dependent	Larger particles favored rutting-related response and fatigue; smaller particles favored low-temperature cracking and storage stability	Confounded with SBS structure
Lei et al. [12]	20, 40, 80, 100 mesh (0.850, 0.425, 0.180, 0.150 mm); bio-oil pretreated	High-temperature elasticity, fatigue, storage stability	Finer favored for fatigue; mid-range for storage	Predicted fatigue life rose with increasing mesh number, that is with decreasing size, the finest fraction (100 mesh) giving the longest predicted life; storage stability was optimal at a mid-range 40 mesh, with 80 mesh close to it. The abstract of that paper refers to mesh number rather than to particle diameter, which is why it reads as the opposite	Confounded with bio-oil pretreatment
Wang et al. [9]	Finer vs. coarser CRM	High-temperature viscosity	Finer favored	Finer CRM produced higher high-temperature viscosity, consistent with stronger asphalt–rubber interaction	Isolated
Guo et al. [71]	Finer vs. coarser CRM fractions, with SBS co-modification	High- and low-temperature response; aging	Finer favored before aging; effect changed after aging	Finer fractions improved response initially; coarser particles became more influential after aging	Confounded with SBS structure
Cai et al. [64]	Small/medium vs. large fractions, separated by thermal filtration	J_nr_, fatigue, high-temperature deformation	Property-dependent	Small and medium particles reduced J_nr_ and improved fatigue; larger particles favored high-temperature deformation resistance	Isolated (size distribution measured)
Liu et al. [37]	60 vs. 80 mesh (0.250 vs. 0.180 mm)	Binder response	Limited difference	Closely spaced fine fractions behaved similarly, suggesting limited sensitivity across a narrow size interval	Isolated

**Table 7:** Experimental studies on crumb-rubber particle size and binder storage stability, with the storage protocol and separation index stated for each. Studies are grouped by the type of separation index, because indices of different type are not directly comparable. All nine protocols were read from the primary sources. Two features of the record bear on comparability. The oven stage is close to uniform, 163 °C for 48 h in eight of the nine studies, whereas the temperature at which the tube is chilled before sectioning ranges from 10 ± 10 °C to −18 °C among studies that cite the same ASTM D7173 [82] procedure. Two studies measure separation without sectioning at all, one by sampling at fixed heights and one by tomography. Where a source does not state a condition, that is said in the cell rather than left blank. G* denotes the complex shear modulus. The ASTM D7173 edition designations shown are those reported by the individual source studies.

Study	Group	CRM Size (As Charged)	Binder/ Dosage	Storage Protocol	Sectioning and Index	Key Finding
Willis et al. [36]	Softening-point index	Eleven products, varied mean size and grinding method	PG 67-22; 10% (two products also at 15%)	ASTM D7173-11 [82]; 50 g in a sealed ¾-in. aluminum tube; 163 ± 5 °C for 48 ± 1 h, static (no agitation); blended at 163 °C, 1000 rpm, 30 min	Frozen at 10 ± 10 °C for ≥4 h, cut into three equal portions with the middle discarded; ΔSP (ASTM D36/D36M-09) [83] between top and bottom, plus critical high-temperature grade by DSR (1 mm gap; 2 mm for the coarsest blend)	Smallest-size product showed the least separation under the tested conditions
Lei et al. [12]	Softening-point index	20, 40, 80, 100 mesh (0.850–0.150 mm); bio-oil pretreated	80–100 pen; composite modifier	Cigar-tube test under a static heated condition; glass tube 15 mm diameter × 100 mm length sealed with a rubber block against air; held vertical in an oven, 163 °C for 48 h	Cooled to ambient, then frozen 4 h at −18 °C; cut into three equal portions, top and bottom each stirred to a representative specimen; segregation index from the rut factor (G*/sinδ) of a temperature sweep at 52 °C	Lowest segregation index at 40 mesh, with 80 mesh close to it and 20 mesh the highest; the authors caution that the index is a reference for the conditions tested rather than a general ranking; predicted fatigue life increased as the rubber became finer, the finest fraction (100 mesh) giving the longest life
Navarro et al. [10]	Rheological index	Five fractions, mean 0.10, 0.29, 0.35, 0.63 and 0.74 mm, screened from a 1 mm commercial ground rubber	60/70 pen; 9 wt%	Static storage test; tube 3.2 cm diameter × 40 cm height, held vertical in an oven; 150 and 180 °C for 12 h, no agitation; blended at 180 °C for 1.5 h in an open low-shear four-blade impeller, 1200 rpm	No sectioning: sampled at four heights (0 (bottom), 10, 20 and 30 cm); frequency sweep at 50 °C, and a stability index defined as the ratio of the zero-shear-limiting viscosity at that height to the same quantity on a fresh, unstored sample (index near unity denotes a stable binder). Softening point was not measured in this study	Stability decreased with increasing size; stable at or below 0.29 mm under the conditions tested, and sizes below 0.35 mm with high shear during manufacture recommended
Vigneswaran et al. [13]	Rheological index	≤0.5 mm, ≤1 mm, 1–2 mm (ambient mechanical milling)	PG 64-22; 10%	ASTM D7173; 50 ± 0.5 g in an upright aluminum cigar tube; 163 ± 5 °C for 48 h in a drying oven, static; stirred manually before pouring; blended at 178 ± 2 °C, 700 rpm (low shear), 30 min	Cooled ≥ 4 h at 10 ± 10 °C; cut into three equal sections (top, middle, bottom), remelted at 163 ± 5 °C and stirred; SI from G*/sinδ and percent recovery (DSR/MSCR)	SI increased with particle size; ≤0.5 mm most stable
Yun et al. [78]	Rheological index	No. 50 vs. No. 30 mesh (0.300 vs. 0.600 mm)	PG 64-22; 5% and 15%	ASTM D7173; 50 ± 0.5 g in a sealed upright aluminum tube; 163 ± 5 °C for 48 h, static; stirred before pouring; blended at 177 °C, 700 rpm (open blade), 30 min	Frozen ≥ 4 h at −10 ± 10 °C held vertical; cut into three equal sections, casing removed at 163 ± 5 °C within 30 min; SI from G*/sinδ and percent recovery, both at 64 °C; AFM (tapping mode, 20 × 20 µm)	Finer fraction gave lower SI and a more uniform top/bottom microstructure
Liang et al. [43]	Rheological index	Four mean diameters: 0.15, 0.18, 0.25 and 0.42 mm (ambient-ground)	Two penetration-grade asphalts; 9 wt%	Chinese specification JTJ F40 [43]; standard aluminum tube 2.5 cm diameter × 14 cm height, sealed and held vertical in an oven at 163 °C for 48 h, static; blended 3 h at 170 °C in a four-blade impeller, 1200 rpm	Cooled in a refrigerator (temperature not stated); divided into three equal sections; frequency sweep at 50 °C on the upper and bottom sections, comparing G′ and G″ and the zero-shear viscosity from the discrete relaxation spectrum	The top-bottom difference narrowed as size decreased, so smaller particles gave the better storage stability; smaller particles were also less distinguishable within the matrix
Ghavibazoo et al. [51]	Rheological index	0.40–0.55 mm (No. 40–No. 30); dissolution level varied	PG 58-28	Interaction at 190 °C and 220 °C; liquid-phase viscosity measured at 163 °C	Standard storage-stability index; Stokes’-law analysis	Dissolution improves stability at 190 °C through residual size reduction and liquid-phase viscosity growth; the separation mechanism changes at 220 °C
Cao et al. [79]	Tomographic index	10–60 mesh (2.00–0.250 mm), ambient-ground	PEN 70	Oven at 163 °C for 0–48 h, static (a time series rather than a single hold); specimens cast in carbon-fiber square-tube moulds and cured 4 h at room temperature; blended at 180 °C, 1100 rpm, 30 min. A parallel cigar-tube hold at 163 °C for 48 h supplied the reference ΔSP	No sectioning—X-ray CT is non-destructive; the specimen is divided computationally into three equal segments along its height and the midpoint of each is analyzed. CR area ratio (two-dimensional) and CR volume ratio (three-dimensional, outer 2 mm edge excluded); ΔSP between top and bottom measured separately as the reference	Separation increased with size (10 mesh: 25.9% top-bottom difference in area ratio; 60 mesh: 9.2%), and migration was largely complete by 18 h; R^2^ > 0.75 between the rate of volume change and particle size. The CT indices were validated against ΔSP (correlation about 80%) rather than offered in place of it
Hosseinnezhad et al. [34]	Surface-treatment comparison (size not varied)	Surface-activated rubber; size not the variable	Base binder with CRM	ASTM D7173-14 [82] cigar-tube separation test; poured at 163 °C into aluminum tubes held vertical in a rack with the tops sealed against air intrusion; oven at 163 °C for 48 h, static	Rack frozen 4 h at −18 °C; cut into three equal sections with the middle discarded; DSR at 58 °C on the top and bottom sections; SI from complex modulus and phase angle	Surface functionalization substantially reduced phase separation; not a particle-size comparison

**Table 8:** Effect of decreasing crumb-rubber particle size on the binder responses within the scope of this review. Entries state the direction reported in the cited sources and the interpretation offered here; where the direction is conditional, this is stated rather than resolved. G* denotes the complex shear modulus; Jnr, non-recoverable creep compliance.

Binder Response	Effect of Finer CRM	Interpretation	Evidence
High-temperature rutting-related binder response (G*/sinδ, MSCR J_nr_)	System-dependent; may improve with fine-to-intermediate CRM under some conditions	Depends on the balance between particle reinforcement and asphalt–rubber interaction	[9,10]
Predicted binder fatigue life	System-dependent, and the two sources that report it disagree: finer favored in [12], coarser in [70]	Not isolated experimentally in either source. Ref. [70] attributes the advantage of the coarser fractions to retention of a discrete elastic inclusion, whereas [12] attributes the advantage of the finer fractions to more complete depolymerization, crack propagation being localized around incompletely digested particles. LAS in [12]; rheological characterization in [70]	[12,70]
Storage stability	Generally improves	Finer CRM reduces phase separation, with magnitude depending on processing and interaction conditions	[10,13,36,79]
Rotational viscosity	System-dependent; may increase	Depends on interaction state, particle effect, processing conditions, and test configuration	[9,20,86]
Workability	System-dependent	Follows viscosity, residual particle size, interaction state, CRM content, processing conditions, and test temperature	[9,20]

**Table 9:** Study-specific favorable particle sizes, expressed on a common metric basis. Mesh values are converted using ASTM E11 (Table 4). The “Basis” column records which size descriptor the source uses, because upper-bound, retained-fraction, and mean-diameter values are not interchangeable (Section 2.6). The final column states whether the reported favorable size lies within the 0.180–0.425 mm cluster discussed in Section 7.1.

Study	Sizes Investigated	Favorable Finding	Metric Equivalent	Size Descriptor	Criterion	Within 0.180–0.425 mm?
Cao et al. [80]	20, 40, 60, 80, 120 mesh	80 mesh	0.180 mm	Retained fraction	Overall binder response	Yes (lower bound)
Lei et al. [12]	20, 40, 80, 100 mesh	40 mesh, storage stability only	0.425 mm	Retained fraction	Storage stability; the predicted-fatigue-life optimum in that study was the finest fraction tested, 100 mesh, which falls below the interval	Yes (upper bound)
Zhang et al. [67]	28, 40, 60 mesh	40 mesh	0.425 mm	Retained fraction	Overall rheological behavior	Yes (upper bound)
Liu et al. [37]	60, 80 mesh	60 and 80 behaved similarly; no optimum identified	0.250 and 0.180 mm	Retained fraction	Binder response	Both inside, but reports no optimum
Navarro et al. [10]	Mean sizes 0.10–0.74 mm	<0.35 mm with high-shear mixing	<0.35 mm	Mean diameter	Storage stability	Overlaps; upper bound falls inside
Vigneswaran et al. [13]	Fractions reported in mm	≤0.5 mm favored	≤0.5 mm	Passing size (upper bound)	Storage stability; 1–2 mm gave a more favorable rutting-related response but poorer workability	No—extends coarser than 0.425 mm

## Data Availability

No new data were created or analyzed in this study. Data sharing is not applicable.

## Supplementary Materials

The following supporting information can be downloaded at: https://www.mdpi.com/article/10.3390/polym18182283/s1, Table S1: Included sources—the 83 sources retained in this review, with source type, the study-level table in which each is tabulated, and whether an abstract or full text was obtainable for citation-accuracy checking.

## Abbreviations

AFM, atomic force microscopy; ASTM, ASTM International; BBR, bending beam rheometer; BET, Brunauer–Emmett–Teller (specific surface area); CRM, crumb rubber modifier; CT, computed tomography; DSC, differential scanning calorimetry; DSR, dynamic shear rheometer; ELT, end-of-life tire; FTIR, Fourier-transform infrared spectroscopy; GPC, gel permeation chromatography; GTR, ground tire rubber; J_nr_, non-recoverable creep compliance; LAS, linear amplitude sweep; MSCR, multiple stress creep and recovery; PG, performance grade; PRISMA, Preferred Reporting Items for Systematic Reviews and Meta-Analyses; RTR, recycled tire rubber; SBS, styrene-butadiene-styrene; SEM, scanning electron microscopy; SI, separation index; ΔSP, softening-point difference; TRID, Transport Research International Documentation; VECD, viscoelastic continuum damage.
