# Supplementary material for: Effect of Crumb Rubber Particle Size on the Rheological Response, Fatigue Response, and Storage Stability of Asphalt Binders: A Review
Source: Polymers (Basel). 2026 Sep 18;18(18):2283. doi: 10.3390/polym18182283 (PMC13611380; doi:10.3390/polym18182283)
Supplement: Supplementary file 1 [file polymers-18-02283-s001.zip › polymers-4564117-supplementary.pdf]

Supplementary Table S1 — Included sources

Manuscript: polymers-4564117 · Effect of Crumb Rubber Particle Size on the Rheological Response, Fatigue Response, and Storage Stability of Asphalt Binders: A Review

The 83 sources retained in this review, provided in response to Reviewer 1 Comment 1 and Reviewer 2 Comment 2.

How to read this table

- Source type is determined from the bibliographic record: *Journal article*, *Review*, *Conference*, or *Report*.
- In tables shows where the study-level extraction appears: T6 conflict matrix (reported size effects and whether size was isolated experimentally), T7 storage stability (storage protocol and separation index), T9 favorable sizes (metric equivalent, size descriptor, criterion). A dash means the study is discussed in the text but is not tabulated.
- Abstract source records where the text used for citation-accuracy checking was obtained: *OpenAlex* or *S2* for an abstract, *full text* where the full article was obtained and read, and *not obtained* where neither was available from Crossref, OpenAlex, or Semantic Scholar and no accessible copy could be found. The 32 sources marked *not obtained* are predominantly subscription-only Elsevier titles.

On the primary/supporting-context distinction. Section 2.3 states the rule by which mixture-scale and specification-oriented studies are treated as supporting context rather than primary evidence. That rule applies at the point of citation, not to a study as a whole — the same source can supply primary binder-scale evidence in one section and context in another. It is therefore applied in the text and in Tables 6, 7 and 9, and is not asserted per row here.

> Detailed extraction fields are reported for the 17 sources that directly compare particle sizes and underpin Tables 6, 7, or 9. The remaining sources support background, mechanisms, methods, or contextual interpretation and are listed bibliographically here. Because full study-level extraction was not completed for every contextual source, this supplement should not be interpreted as a complete extraction matrix for all 83 sources.

Summary	Count
Retained sources	83
— of which journal article	68

Summary	Count
— of which review	11
— of which conference	2
— of which report	2
Tabulated in T6 / T7 / T9	17
Source obtained for citation-accuracy checking	51
— of which abstract only	38
— of which full text	13
No source obtainable	32
Of the 51 checked — supported by the source	48
Of the 51 checked — partly supported	2
Of the 51 checked — source did not address the statement	1

Study list

Ref	First author	Year	DOI	Source type	In tables	Abstract source
1	Karaağaç	2017	10.1007/s10163-015-0427-2	Journal article	—	full text
2	Grammelis	2021	10.3390/en14030571	Review	—	OpenAlex
3	Hassan	2024	10.3390/su16093852	Journal article	—	full text
4	Ibrahim	2013	10.1155/2013/415246	Review	—	OpenAlex
5	Lo	2013	10.1016/j.conbuildmat.2013.09.007	Review	—	OpenAlex
6	Abdelrahman	1999	10.3141/1661-15	Journal article	—	OpenAlex
7	Putman	2006	—	Conference	—	not obtained
8	Singh	2018	10.1061/(ASCE)MT.1943-5533.0002245	Journal article	—	OpenAlex
9	Wang	2012	10.1016/j.conbuildmat.2011.06.0	Journal article	T6	not obtained

Ref	First author	Year	DOI	Source type	In tables	Abstract source
			61			
10	Navarro	2004	10.1016/j.fuel.2004.04.003	Journal article	T7, T9	full text
11	Shen	2009	10.1016/j.conbuildmat.2007.12.005	Journal article	—	not obtained
12	Lei	2021	10.1016/j.conbuildmat.2021.122864	Journal article	T6, T7, T9	full text
13	Vigneswaran	2023	10.3390/su151813568	Journal article	T7, T9	full text
14	Jeong	2010	10.1016/j.conbuildmat.2009.10.024	Journal article	—	not obtained
15	Thodesen	2009	10.1016/j.conbuildmat.2007.12.007	Journal article	—	not obtained
16	Chiu	2007	10.1016/j.conbuildmat.2006.02.005	Journal article	—	not obtained
18	Zakerzadeh	2024	10.1016/j.conbuildmat.2024.135420	Review	—	OpenAlex
19	Bennert	2013	—	Report	—	not obtained
20	Ding	2020	10.1016/j.conbuildmat.2020.118454	Journal article	—	S2
21	Bressi	2019	10.3390/coatings9060384	Review	—	full text
22	Wang	2017	10.1016/j.conbuildmat.2017.04.031	Review	—	not obtained
23	Zheng	2021	10.1016/j.conbuildmat.2021.123820	Review	—	S2
24	Duan	2022	10.1016/j.conbuildmat.2022.129687	Journal article	—	not obtained
25	Yang	2024	10.1016/j.jclepro.2024.141595	Review	—	not obtained

Ref	First author	Year	DOI	Source type	In tables	Abstract source
26	Lyu	2024	10.1016/j.jclepro.2023.140202	Review	—	OpenAlex
28	Loderer	2018	10.1016/j.conbuildmat.2018.10.046	Journal article	—	S2
29	Lee	2008	10.1016/j.conbuildmat.2007.04.010	Journal article	—	not obtained
30	Pais	2019	10.1016/j.conbuildmat.2019.03.155	Journal article	—	full text
31	Heitzman	1992	—	Journal article	—	full text
32	Medina	2020	10.1016/j.conbuildmat.2019.117351	Journal article	—	S2
33	Xu	2022	10.1016/j.jclepro.2021.130113	Journal article	—	not obtained
34	Hosseinnezhad	2019	10.1016/j.jclepro.2019.03.219	Journal article	T7	full text
35	Yang	2020	10.1016/j.jclepro.2019.119230	Journal article	—	S2
36	Willis	2012	—	Report	T7	full text
37	Liu	2009	10.1016/j.conbuildmat.2008.12.009	Journal article	T6, T9	not obtained
38	Ren	2021	10.1016/j.conbuildmat.2021.124966	Journal article	—	OpenAlex
39	Rath	2021	10.1177/03611981211011480	Journal article	—	OpenAlex
40	Huang	2017	10.1016/j.conbuildmat.2017.03.229	Journal article	—	not obtained
41	Wen	2020	10.1016/j.conbuildmat.2020.119586	Review	—	S2
42	Ghavibazoo	2013	10.3141/2370-12	Journal article	—	OpenAlex
43	Liang	2015	10.1016/j.conbuildmat.2014.10.015	Journal article	T7	full text

Ref	First author	Year	DOI	Source type	In tables	Abstract source
44	Jamal	2021	10.1016/j.conbuildmat.2021.124638	Journal article	—	not obtained
45	Bahia	1994	—	Journal article	—	not obtained
46	Peralta	2012	10.1016/j.conbuildmat.2012.06.030	Journal article	—	not obtained
47	Santagata	2025	10.1016/j.conbuildmat.2024.139573	Journal article	—	OpenAlex
48	Airey	2003	10.1080/1029843032000158879	Journal article	—	OpenAlex
49	Frantzis	2004	10.1061/(ASCE)0899-1561(2004)16:4(387)	Journal article	—	OpenAlex
50	Farouk	2017	10.1617/s11527-016-0932-3	Journal article	—	not obtained
51	Ghavibazoo	2013	10.3141/2370-14	Journal article	T7	OpenAlex
52	Ren	2022	10.1016/j.conbuildmat.2022.127555	Journal article	—	OpenAlex
53	Xing	2023	10.1016/j.conbuildmat.2022.130262	Journal article	—	not obtained
54	Zheng	2026	10.1016/j.jclepro.2026.147959	Journal article	—	not obtained
55	Ragab	2018	10.1016/j.ijprt.2017.08.003	Journal article	—	OpenAlex
56	Wang	2022	10.1177/03611981221088595	Journal article	—	OpenAlex
57	Liu	2025	10.1016/j.conbuildmat.2025.144548	Journal article	—	not obtained
58	Tabatabaee	2010	10.3141/2180-08	Journal article	—	OpenAlex
59	Navarro	2002	10.1016/S1385-8947(02)00023-2	Journal article	T6	not obtained
60	Thodesen	2009	10.1016/j.conbuildmat.2009.04.005	Journal article	—	not obtained
61	Xiao	2011	10.1080/10298430903578903	Journal article	—	OpenAlex

Ref	First author	Year	DOI	Source type	In tables	Abstract source
62	Zhu	2022	10.1016/j.conbuildmat.2022.127488	Journal article	—	not obtained
63	Qiao	2025	10.1016/j.conbuildmat.2025.142129	Journal article	—	not obtained
64	Cai	2025	10.1016/j.cscm.2025.e04594	Journal article	T6	OpenAlex
65	Xie	2022	10.3389/fmats.2022.904087	Journal article	—	OpenAlex
66	Shenoy	2001	10.1061/(ASCE)0733-947X(2001)127:5(357)	Journal article	—	OpenAlex
67	Zhang	2025	10.1016/j.conbuildmat.2025.140520	Journal article	T9	not obtained
68	Gui	2021	10.1016/j.conbuildmat.2021.122881	Journal article	—	S2
69	Al-Khateeb	2024	10.3390/polym16070906	Journal article	—	OpenAlex
70	Qian	2020	10.1016/j.conbuildmat.2019.117517	Journal article	T6	S2
71	Guo	2024	10.1016/j.cscm.2024.e03232	Journal article	T6	OpenAlex
72	Zhou	2024	10.1016/j.jclepro.2024.142168	Journal article	—	not obtained
73	Nan	2022	10.1016/j.conbuildmat.2022.129651	Journal article	—	not obtained
74	Li	2019	10.1016/j.conbuildmat.2019.03.136	Journal article	—	S2
75	Wang	2025	10.1016/j.conbuildmat.2025.141801	Journal article	—	not obtained
76	Venudharan	2018	10.1016/j.conbuildmat.2018.06.087	Journal article	—	S2
77	Sienkiewicz	2017	10.1016/j.fuproc.2017.01.049	Review	—	not obtained

Ref	First author	Year	DOI	Source type	In tables	Abstract source
78	Yun	2023	10.22702/JKAI.2023.13.2.30	Journal article	T7	full text
79	Cao	2026	10.1016/j.fuel.2025.136955	Journal article	T7	full text
80	Cao	2011	10.4028/www.scientific.net/AMM.99-100.955	Journal article	T9	OpenAlex
81	Wang	2020	10.1016/j.conbuildmat.2020.118840	Journal article	—	OpenAlex
85	Liu	2022	10.1016/j.conbuildmat.2021.125676	Journal article	—	not obtained
86	Dantas	2006	—	Conference	—	OpenAlex
87	Venudharan	2017	10.1617/s11527-017-0994-x	Journal article	—	not obtained
88	Venudharan	2017	10.1061/(ASCE)MT.1943-5533.0001880	Journal article	—	OpenAlex

Verification note

All 83 references are cited in the text and the numbering follows order of first appearance. Absence of uncited entries, absence of citations to missing entries, and first-appearance ordering were checked mechanically for the revised manuscript.

Separately, each reference was checked against its source abstract for whether the cited work supports the statement made. This was carried out in two passes. In the first, 45 sources were obtainable and six citations were corrected. Because this revision rewrote Section 2 in full, expanded the tables from five to nine, and reframed the 40-80 mesh interval, the citing sentences changed for 59 of the 83 references; the 28 of those that had previously been judged supported were therefore re-checked against freshly retrieved abstracts, and 17 passages across 11 references were corrected as a result. The corrections were of three kinds: statements whose mechanism did not match the cited abstract (three references), co-citations in which one of the two sources did not support the specific figure or claim (three references), and test names or evaluation scales attributed more precisely than the abstract warranted (five references).

A third pass followed, in which the full texts of eight references were obtained and read; five of these were subscription-only titles for which no abstract had been available. The current status of the 83 references is therefore as follows. A source — an abstract or the full text — was obtained for 51 references, and no source could be obtained for the remaining 32. Of the 51, 48 are supported by the

source and 2 are partly supported; for 1 further reference the source was obtained but proved not to address the statement it had been cited for, and that statement has been narrowed. None of the 83 is contradicted by its source. The two partly supported entries are identified in the text where they occur. The 32 references for which no source could be obtained were not altered, and the text states where the underlying evidence is limited.
